# Antibody-dependent cellular cytotoxicity, infected cell binding and neutralization by antibodies to the SIV envelope glycoprotein

**DOI:** 10.1371/journal.ppat.1011407

**Published:** 2023-05-30

**Authors:** Michael W. Grunst, Ruby A. Ladd, Natasha M. Clark, Hwi Min Gil, Vadim A. Klenchin, Rosemarie Mason, Genoveffa Franchini, Mario Roederer, David T. Evans

**Affiliations:** 1 Department of Pathology and Laboratory Medicine, University of Wisconsin-Madison, Madison, Wisconsin, United States of America; 2 Wisconsin National Primate Research Center, University of Wisconsin-Madison, Madison, Wisconsin, United States of America; 3 Vaccine Research Center, National Institute of Allergy and Infectious Diseases (NIAID), National Institutes of Health (NIH), Bethesda, Maryland, United States of America; 4 Animal Models and Retroviral Vaccines Section, National Cancer Institute, Bethesda, Maryland, United States of America; Emory University, UNITED STATES

## Abstract

Antibodies specific for diverse epitopes of the simian immunodeficiency virus envelope glycoprotein (SIV Env) have been isolated from rhesus macaques to provide physiologically relevant reagents for investigating antibody-mediated protection in this species as a nonhuman primate model for HIV/AIDS. With increasing interest in the contribution of Fc-mediated effector functions to protective immunity, we selected thirty antibodies representing different classes of SIV Env epitopes for a comparison of antibody-dependent cellular cytotoxicity (ADCC), binding to Env on the surface of infected cells and neutralization of viral infectivity. These activities were measured against cells infected with neutralization-sensitive (SIV_mac_316 and SIV_sm_E660-FL14) and neutralization-resistant (SIV_mac_239 and SIV_sm_E543-3) viruses representing genetically distinct isolates. Antibodies to the CD4-binding site and CD4-inducible epitopes were identified with especially potent ADCC against all four viruses. ADCC correlated well with antibody binding to virus-infected cells. ADCC also correlated with neutralization. However, several instances of ADCC without detectable neutralization or neutralization without detectable ADCC were observed. The incomplete correspondence between ADCC and neutralization shows that some antibody-Env interactions can uncouple these antiviral activities. Nevertheless, the overall correlation between neutralization and ADCC implies that most antibodies that are capable of binding to Env on the surface of virions to block infectivity are also capable of binding to Env on the surface of virus-infected cells to direct their elimination by ADCC.

## Author summary

Antibodies to the envelope glycoprotein (Env) of simian immunodeficiency virus (SIV) have been isolated to investigate mechanisms of antibody-mediated protection in rhesus macaques as a nonhuman primate model for HIV-1/AIDS. Antibodies that bind to Env on virus particles can block SIV infectivity. Antibodies that bind to Env on the surface of virus-infected cells can also recruit cells of the immune system to kill infected cells by a process known as antibody-dependent cellular cytotoxicity (ADCC). We compared antibodies to diverse surfaces of Env for their ability to mediate ADCC, bind to SIV-infected cells and block viral infectivity. We found that most antibodies that inhibit SIV infectivity also bind to Env on infected cells and mediate ADCC. However, there were a few instances for which these antiviral functions did not correspond, indicating that certain types of antibodies may uncouple these functional activities. Our findings provide an important conceptual framework and a valuable dataset for investigating antiviral functions of antibodies in macaques as a pre-clinical model for testing antibody-based vaccines and immunotherapies for HIV-1/AIDS.

## Introduction

Recent efforts to develop effective vaccines and immunotherapies for HIV-1 have primarily focused on antibody responses to the viral envelope glycoprotein [1,2]. The most effective antibodies can neutralize genetically diverse HIV-1 field isolates at low concentrations and are referred to as potent broadly neutralizing antibodies (bnAbs). These antibodies are capable of binding to functional Env trimers as they exist on virions to block infectivity. However, most antibodies to Env elicited in response to vaccination or natural infection are non-neutralizing [3,4]. Non-neutralizing antibodies (nnAbs) bind to surfaces of Env that are not accessible prior to receptor engagement and by definition cannot block viral infectivity. However, under certain circumstances these antibodies may contribute to the elimination of virus-infected cells through Fc-dependent mechanisms such as antibody-dependent cellular cytotoxicity (ADCC).

Several correlative studies suggest that Fc-mediated effector functions may contribute to protective immunity. Although ADCC was not identified as a correlate of protection in the RV144 trial, a non-significant trend towards a lower risk of HIV-1 infection was observed among vaccine recipients with higher ADCC responses [5]. Follow-up studies later revealed the induction of Env-specific antibodies among vaccinated subjects of the RV144 trial with enhanced Fcɣ receptor (FcɣR)-mediated functions [6–8]. Higher ADCC responses have also been associated with better clinical outcomes in HIV-infected patients [9–15] and with partial or complete protection against mucosal SIV or SHIV challenge in nonhuman primate models [16–21]. While these observations do not directly implicate ADCC as a protective mechanism, they have prompted a number of animal studies to investigate the contribution of FcɣR-mediated effector functions to protective immunity.

The results of passive antibody transfer experiments to directly assess Fc-mediated protection in nonhuman primate models have been mixed. In support of Fc-mediated protection, Fc domain substitutions in a first-generation HIV-1 bnAb that abrogate FcɣR binding impaired protection against SHIV challenge [22,23]. FcɣR null mutations also reduced the rate of viral load decline following the treatment of SHIV-infected animals with a bispecific bnAb [24]. Passive transfer of purified IgG from vaccinated macaques to naïve animals afforded partial protection against mucosal SIV challenge in the absence of detectable neutralizing antibodies [20]. Furthermore, AAV delivery of an SIV Env-specific antibody capable of mediating ADCC without detectable neutralization protected one animal against mucosal SIV challenge [25]. However, other studies have failed to support these observations. The passive administration of a number of HIV-1 nnAbs, including some reported to mediate ADCC *in vitro*, failed to protect macaques against SHIV challenge [26–30]. The administration of a non-fucosylated HIV-1 bnAb with increased ADCC activity also did not enhance protection against SHIV challenge [31]. Moreover, contrary to earlier results with a first generation bnAb [22,23], Fc domain substitutions in a potent second generation bnAb that abrogate FcɣR binding did not diminish protection against SHIV challenge [32,33].

To facilitate the investigation of antibody-mediated protection in nonhuman primate models, more than 70 antibodies to diverse epitopes of the SIV envelope glycoprotein were isolated from infected or vaccinated rhesus macaques [34,35]. SIV Env-specific antibodies make it possible to assess protection using pathogenic SIV challenge strains that do not require extensive adaptation for efficient replication in animals [36–40]. This is an important advantage over SHIV challenge strains that are often poorly adapted for replication in macaques and have acquired changes in Env that reduce their sensitivity to certain HIV-1 bnAbs [41–46]. Antibodies isolated from rhesus macaques also induce much lower anti-drug antibody (ADA) responses than human or “simianized” antibodies when administered to macaques [35,47], which is essential for studies designed to achieve sustained antibody concentrations *in vivo* [25,35,47–50]. Rhesus macaque antibodies further ensure physiologically relevant interactions with macaque FcɣRs that may differ for animal studies using human antibodies because of species-specific differences in these receptors [51].

In the present study, we selected rhesus macaque antibodies specific for diverse epitopes of the SIV envelope glycoprotein for a comparison of ADCC, Env binding and neutralization. Our results identify antibodies to the CD4 binding site and to CD4-inducible epitopes with especially broad and potent ADCC. We found that ADCC correlates well with Env binding and neutralization, consistent with the notion that most antibodies that are capable of binding to Env on the surface of virions to block infectivity are also capable of binding to Env on virus-infected cells to mediate ADCC. Nevertheless, instances of ADCC in the absence of neutralization and neutralization in the absence of ADCC were observed, indicating differences in some antibody-Env interactions that can uncouple these antiviral functions.

## Results

Thirty SIV Env-specific antibodies were selected for an analysis of their ADCC responses to virus-infected cells and for comparison of ADCC with Env binding and neutralization of viral infectivity. The majority of these antibodies were isolated from rhesus macaques vaccinated with SIV Env immunogens and subsequently challenged with SIV_mac_239, SIV_mac_251 or SIV_sm_E660 [21,34]. Exceptions were 5L7, which was reconstructed from Fab fragments cloned from an animal infected with a gp120 glycosylation mutant of SIV_mac_239 [25,52], and 1.4H, which was isolated from an HIV-2-infected individual [53,54]. Thus, all of the antibodies have the same rhesus macaque IgG1 Fc domain, except 1.4H, which is human IgG1. Since we previously found that human and rhesus macaque IgG1 mediate similar signaling through FcγR3A (CD16a) [51], the human Fc domain of 1.4H is expected to have a minimal effect on the ADCC activity of this antibody. These antibodies bind to different surfaces of Env, including epitopes in the CD4 binding site (CD4bs), V1/V2 loop, V3 loop, high mannose patch (HMP), gp120-gp41 interface, and the membrane proximal external region (MPER) of gp41 [34,55,56]. Each of the antibodies were tested against four different SIV infectious molecular clones (IMCs): SIV_mac_239, SIV_sm_E543-3, SIV_mac_316 and SIV_sm_E660-FL14. SIV_mac_239 and SIV_sm_E543-3 are independently isolated, neutralization-resistant viruses that differ from one another to a similar extent as unrelated HIV-1 field isolates and share only 83% amino acid identity in Env [38,39]. SIV_mac_316 and SIV_sm_E660-FL14 are neutralization-sensitive viruses related to SIV_mac_239 and SIV_sm_E543-3, respectively [57–59]. SIV_sm_E543-3 and SIV_sm_E660-FL14 share 93% amino acid identity in Env, whereas SIV_mac_239 and SIV_mac_316 differ by only nine amino acids (99% identity) in Env [38,39,57,58]. Hence, this study was designed to compare Env binding, ADCC and neutralization across pairs of genetically distinct SIVs that differ in their sensitivity to antibodies.

### Binding to Env on the surface of SIV-infected cells

**Fig 1:**
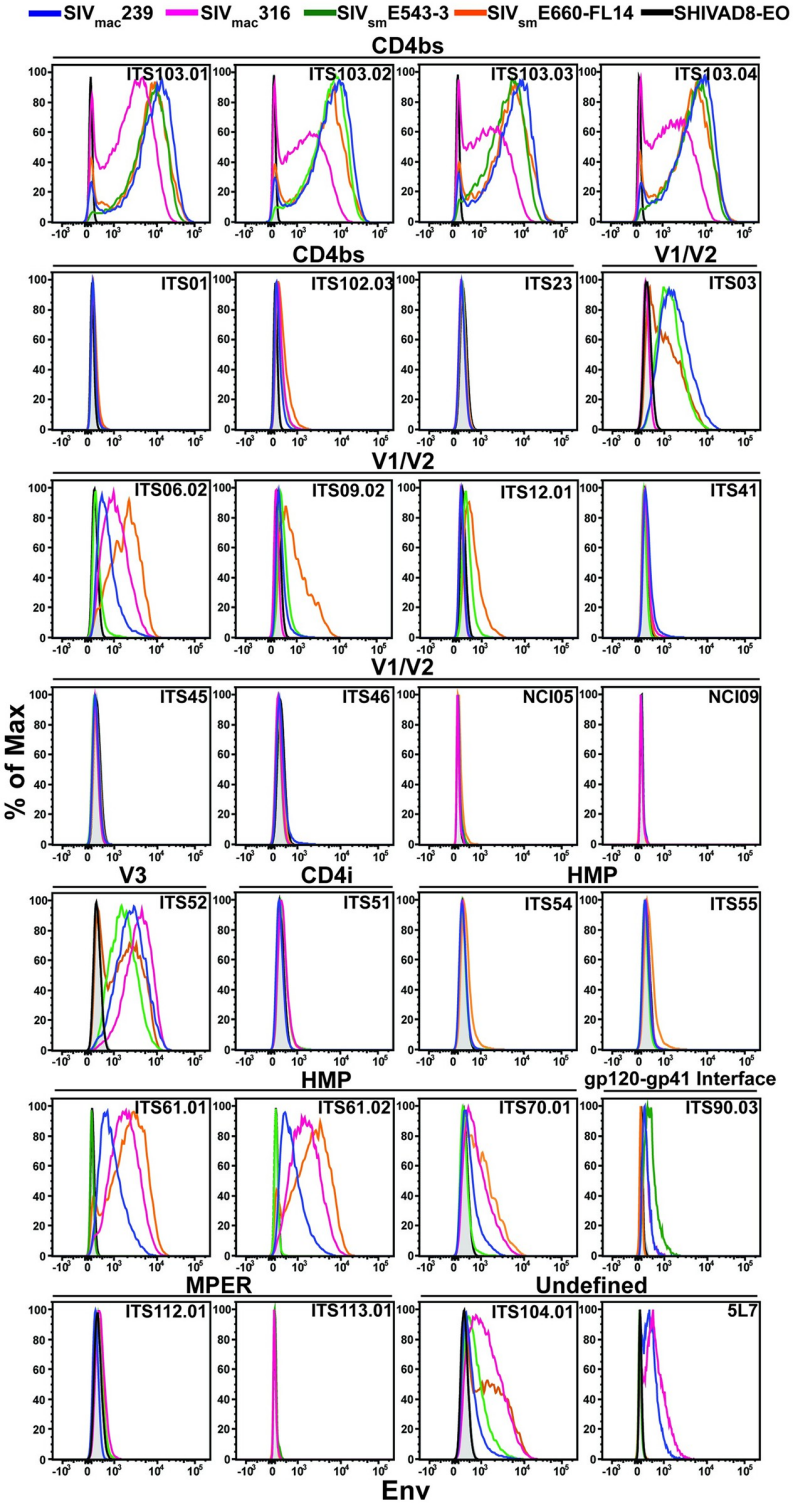
Env staining on the surface of infected CEM.NKR-_CCR5_-sLTR-Luc cells. CEM.NKR-_CCR5_-sLTR-Luc cells were infected with SIV_mac_239, SIV_mac_316, SIV_sm_E543-3, SIV_sm_E660-FL14 and SHIVAD8-EO. After 3–5 days, the cells were stained with the indicated SIV Env-specific antibodies and with a dengue virus-specific antibody (DEN3) as a negative control, followed by AF647-conjugated anti-human IgG F(ab′)_2_. The cells were also stained for surface expression of CD4, intracellular expression of the SIV Gag protein and for viability. The histograms plots depict Env staining in comparison to non-specific staining with DEN3 (shaded) on virus-infected (Gag+ CD4_low_) cells. Representative data is shown from two independent experiments.

**Fig 2:**
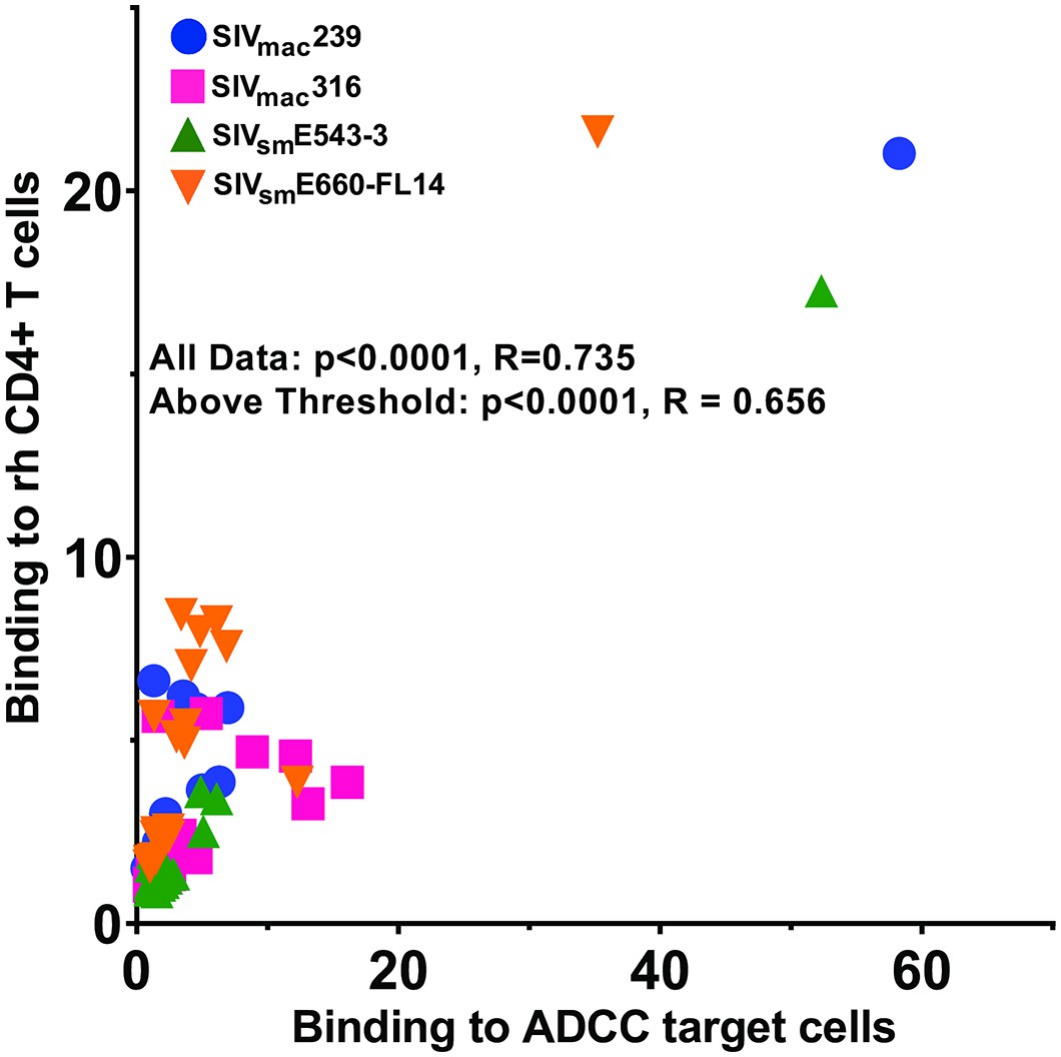
Comparison of Env staining on SIV-infected CEM.NKR-_CCR5_-sLTR-Luc cells and primary rhesus macaque CD4+ T cells. Antibody binding to the surface of SIV-infected cells was quantified as the ratio of the geometric mean fluorescence intensity (gMFI) of staining with each of the Env-specific antibodies to staining with DEN3 control antibody. The relationship between the gMFI ratios of antibody binding to CEM.NKR-_CCR5_-sLTR-Luc cells and to rhesus macaque CD4+ T cells was determined by calculating the Spearman’s rank order correlation.

Antibody binding to Env on the surface of virus-infected cells is a prerequisite for ADCC [60]. We therefore measured the binding of each of the Env-specific antibodies to SIV-infected cells. Env staining was assessed on the surface of the CD4+ T cell line (CEM.NKR-_CCR5_-sLTR-Luc) used as target cells for measuring ADCC (Fig 1) and on activated rhesus macaque CD4+ lymphocytes (S1 and S2 Figs). Although Env staining was generally lower on primary CD4+ T cells than on CEM.NKR-_CCR5_-sLTR-Luc cells, the levels of Env staining on these cells strongly correlated (Fig 2 and S3 Fig). Thus, antibody binding to SIV-infected CEM.NKR-_CCR5_-sLTR-Luc cells reflects antibody binding to virus-infected primary CD4+ T cells.

The highest levels of Env staining were observed for the ITS103 siblings (ITS103.01, ITS103.02, ITS103.03 and ITS103.04), which are clonotypes specific for a conserved epitope in the CD4bs of SIV gp120 [61]. These antibodies bound efficiently to cells infected with all four SIVs, including neutralization-resistant SIV_mac_239 and SIV_sm_E543-3 (Fig 1 and S1 and S2 Figs). However, ITS103 staining was lower on cells infected with neutralization-sensitive SIV_mac_316, consistent with amino acid changes in SIV_mac_316 Env that increase CD4 binding to facilitate infection of macrophages [62]. Broad Env staining was also observed for antibodies specific for V1/V2 (ITS03 and ITS06.02), V3 (ITS52), and HMP (ITS61.01, ITS61.02 and ITS70.01) epitopes, as well as two antibodies of undefined specificity (ITS104.01 and 5L7) (Fig 1 and S1 and S2 Figs). With the exception of ITS03, which bound most efficiently to SIV_mac_239- and SIV_sm_E543-3-infected cells, these antibodies preferentially bound to cells infected with the neutralization-sensitive viruses SIV_mac_316 and SIV_sm_E660-FL14.

### ADCC responses to SIV-infected cells

ADCC was assessed using an assay designed to measure the ability of the antibodies to direct NK cell killing of SIV-infected cells expressing physiologically relevant conformations of the viral envelope glycoprotein [63]. CEM.NKR-_CCR5_-sLTR-Luc cells, which contain an LTR-driven luciferase reporter gene that is inducible by the viral Tat protein, were infected with each of the SIVs and incubated in the presence of serial dilutions of antibody with an NK cell line that constitutively expresses rhesus macaque FcɣR3A (CD16a). ADCC was measured as the dose-dependent loss of luciferase activity after an eight-hour incubation. The rhesus macaque FcɣR3A allele expressed by the NK cell line contains the I158 polymorphism in the extracellular domain, which represents approximately 94% of haplotypes in Indian-origin rhesus macaques [51]. Functional comparisons of the IgG interactions of FcɣR3A I158 with the next most frequent allotype of rhesus macaque FcɣR3A (FcɣR3A V158), which represents approximately 4% haplotypes, indicated that the I158 and V158 polymorphisms exhibit very similar interactions with the human and rhesus macaque IgG subclasses [51].

**Fig 3:**
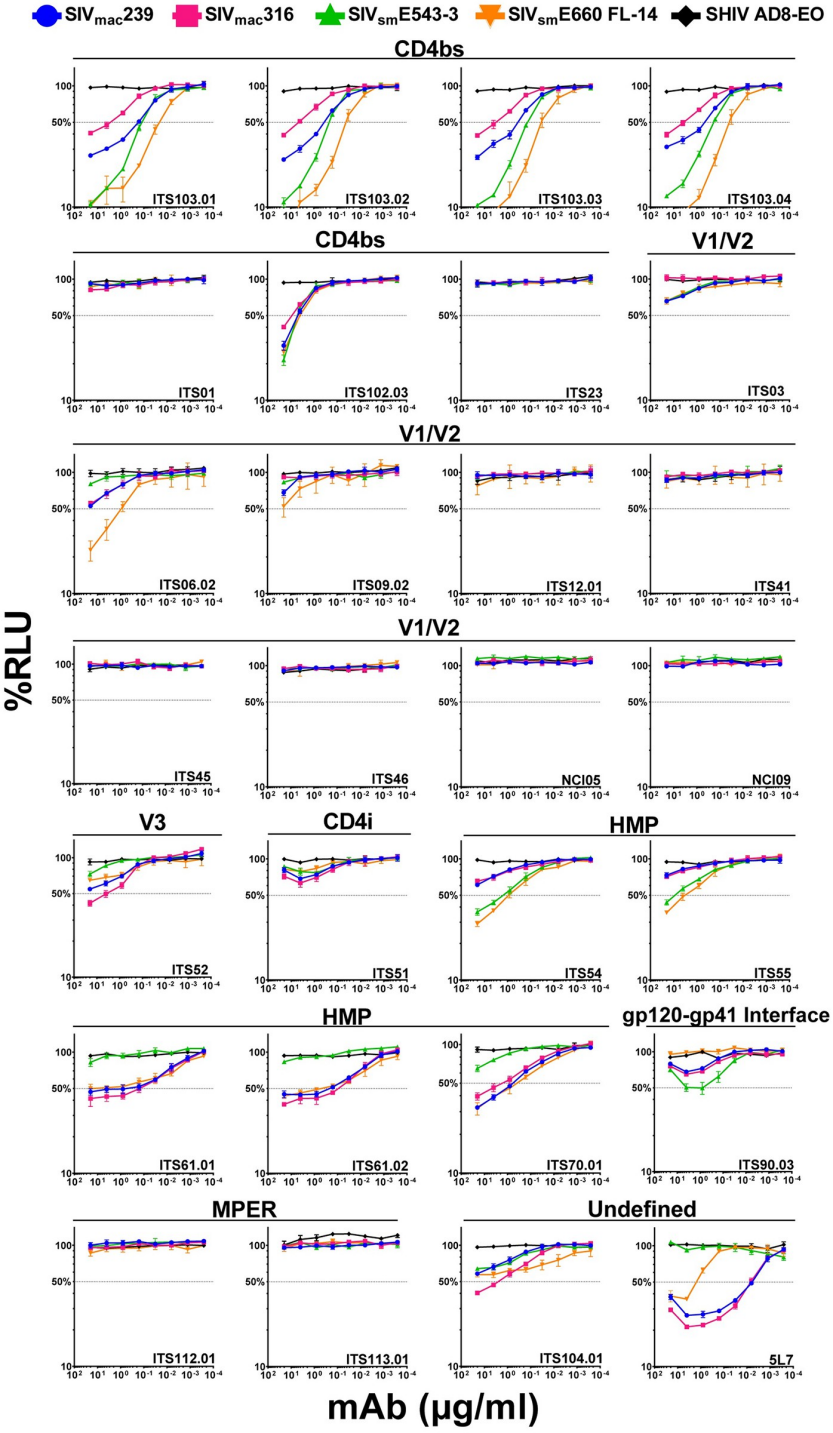
ADCC activity of SIV Env-specific antibodies. CEM.NKR-_CCR5_-sLTR-Luc cells infected with SIV_mac_239, SIV_mac_316, SIV_sm_E543-3, SIV_sm_E660-FL14 and SHIVAD8-EO were incubated with an NK cell line (KHYG-1 cells) expressing rhesus macaque CD16 at a 10:1 effector-to-target (E:T) ratio in the presence of the indicated concentrations of antibody. The dose-dependent loss of luciferase activity was measured in relative light units (RLU) after an 8-hour incubation. ADCC responses were calculated as the remaining luciferase activity (% RLU) by dividing the difference in RLU between SIV-infected cells in the presence of antibody and uninfected cells without antibody (experimental—background) by the difference in RLU between SIV-infected cells and uninfected cells in the absence antibody (maximal–background) and multiplying by 100. The values indicate the mean and standard deviation (error bars) for triplicate wells at each antibody concentration and the dotted line indicates half-maximal killing of SIV-infected cells. Representative data is shown from two independent experiments.

The ADCC responses of the antibodies generally corresponded to their ability to bind to Env on the surface of SIV-infected cells. ITS103 mediated potent ADCC to cells infected with all four SIVs, with lower responses to SIV_mac_316-infected cells in accordance with lower levels Env staining (Fig 3). The ADCC responses of ITS03, ITS06.02, ITS52, ITS61.01/.02, ITS70.1, ITS104.01 and 5L7 also reflected differences in Env staining (Fig 3). In each case where Env staining was detectable on cells infected with neutralization-resistant SIV_mac_239 and SIV_sm_E543-3, ADCC responses were also observed against these viruses. However, the levels of Env staining were not always commensurate with ADCC. Despite marginal levels of Env staining for the CD4bs antibody ITS102.03 and the HMP-specific antibodies ITS54 and ITS55, these antibodies mediated detectable ADCC (Fig 3). ITS102.03 directed similar ADCC responses to cells infected with all four SIVs, whereas ITS54 and ITS55 preferentially killed target cells infected with SIV_sm_E543-3 and SIV_sm_E660-FL14. In contrast, the V2-specific antibodies NCI05 and NCI09 did not exhibit detectable ADCC (Fig 3). Since these antibodies were recently reported to mediate ADCC against gp120-coated cells and to compete with vaccine-induced antibodies associated with a reduced risk of mucosal SIV_mac_251 acquisition [21], they were also tested for binding to gp120-coated cells. NCI05 and NCI09 readily bound to cells coated with SIV_mac_239 gp120 or with wild-type or V1-deleted SIV_mac_251-M766 gp120 (S4 Fig). However, with the exception of a slight shift in NCI05 staining on SIV_sm_E660-FL14-infected cells, these antibodies did not bind to virus-infected cells (Fig 1). These results imply that there are conformational differences in the exposure of the V2 epitopes for NCI05 and NCI09 on monomeric gp120 compared to Env trimers on the surface of SIV-infected cells.

To further investigate the relationship between Env binding and ADCC, we compared ADCC responses to Env staining on virus-infected cells. For ADCC, area above the curve (AAC) values were calculated to provide a consistent measure of responses for all antibodies whether or not they reached an arbitrary threshold of killing (e.g. 50% RLU). AAC values were calculated from the differences between the theoretical maximal luciferase induction (100% RLU) and the percent reductions in luciferase activity at each antibody concentration. For Env binding, the intensity of infected cell staining with each of the Env-specific antibodies was divided by non-specific staining with the DEN3 control antibody to calculate relative Env binding ratios. To avoid biasing comparisons with repeated measures of closely related antibodies, data from the four ITS103 and two ITS61 siblings were averaged and treated as single antibodies.

**Fig 4:**
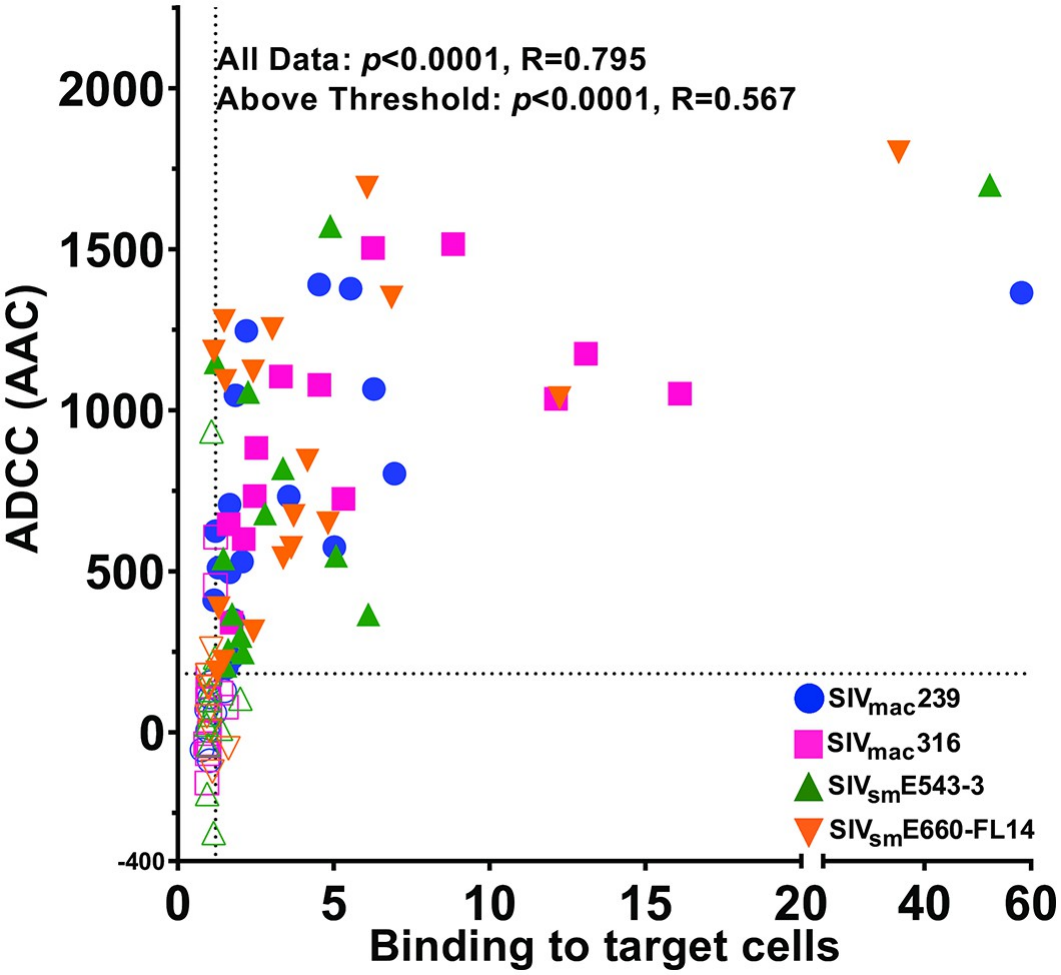
Comparison of ADCC and antibody binding to SIV-infected cells. Area above the curve (AAC) values for ADCC were calculated from differences between the theoretical maximal luciferase induction and the reduction in luciferase activity at each antibody concentration. Antibody binding to SIV-infected CEM.NKR-_CCR5_-sLTR-Luc cells was quantified by calculating the gMFI ratios of staining with each of the Env-specific antibodies to non-specific staining with the DEN3 control antibody. The dotted lines indicate thresholds for detectable ADCC and antibody binding, which were set at one standard deviation above the absolute value of mean of negative ADCC (AAC) values and one standard deviation above the mean of gMFI ratios of antibody binding less than one. Spearman’s rank order correlation coefficients were calculated for comparisons of ADCC and antibody binding for all antibodies and for those antibodies that were above the thresholds for detectable ADCC and binding.

ADCC generally correlated with Env binding (*p*<0.0001, R = 0.795). However, this relationship was complicated by a number of antibodies with little or no Env binding or ADCC (Fig 4 and S1 File). We therefore established a threshold for detectable ADCC at one standard deviation above the absolute value of the mean of the negative values, and a threshold for detectable Env binding at one standard deviation above the mean of gMFI ratios less than one. By these criteria, most of the antibodies that fell below the threshold of detectable binding also fell below the threshold of detectable ADCC. This suggests that a certain level of Env binding is necessary for antibodies to reliably mediate ADCC. With the exclusion of antibodies that lack detectable Env staining or ADCC, the relationship between Env binding and ADCC remained strong (*p*<0.0001, R = 0.567) (Fig 4). However, a wide range of ADCC responses were observed within a similar range of Env binding, indicating a non-linear relationship between these activities. These results suggest that while a certain threshold of antibody binding is required for ADCC, other factors also determine the efficiency of ADCC.

### Neutralization versus ADCC

**Fig 5:**
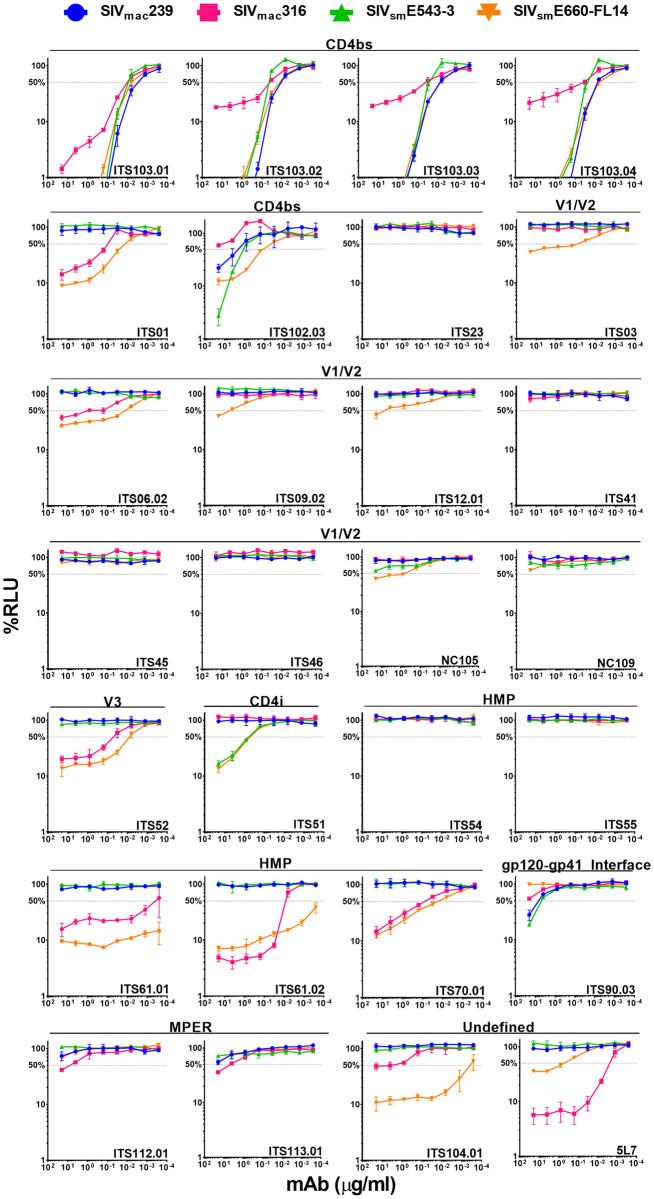
Neutralization of SIV_mac_239, SIV_mac_316, SIV_sm_E543-3 and SIV_sm_E660-FL14 by Env-specific antibodies. Dilutions of each virus were prepared and incubated with the indicated antibody concentrations for one hour before addition to TZM-bl cells. Three days post-infection, neutralization was calculated from the luciferase activity (RLU) in lysates from cells inoculated with virus plus antibody relative to cells inoculated with virus without antibody. The error bars indicate standard deviations of the mean for triplicate wells and the dotted line indicates 50% neutralization.

Although neutralization has previously been reported for many of the SIV Env-specific antibodies [25,34,64], we performed neutralization assays with our IMCs to obtain an internally consistent dataset for comparison with ADCC. Neutralization was measured by the ability to block SIV infection of TZM-bl cells as previously described [65,66]. Each of the ITS103 siblings potently neutralized all four SIVs, but again showed lower activity against SIV_mac_316 (Fig 5). Another CD4bs antibody with broad ADCC activity, ITS102.03, also broadly neutralized SIV_mac_239, SIV_sm_E543-3 and SIV_sm_E660-FL14, but not SIV_mac_316 (Fig 5). Several of the other antibodies that mediated ADCC were able to block SIV infectivity, including ITS03, ITS06.02, ITS52, ITS61.01/.02, ITS70.1, ITS104.01 and 5L7 (Fig 5). However, in each case these responses were only detectable against the neutralization-sensitive IMCs SIV_mac_316 and SIV_sm_E660-FL14 (Fig 5).

Instances of neutralization in the absence of ADCC and ADCC in the absence of neutralization were also observed. Although the CD4bs antibody ITS01 potently neutralized SIV_mac_316 and SIV_sm_E660-FL14, this antibody did not mediate detectable ADCC against cells infected with these viruses (Fig 3). Likewise, ITS112.01 and ITS113.01, which bind to the MPER of gp41, neutralized one or more of the SIVs (Fig 5), but mediated little or no ADCC against cells infected with these viruses (Fig 3). Conversely, the HMP-specific antibodies ITS54 and ITS55 did not neutralize any of the SIV IMCs (Fig 5), but exhibited potent ADCC against cells infected with SIV_mac_316 and SIV_sm_E660-FL14 as well as detectable ADCC to cells infected with SIV_mac_239 and SIV_sm_E543-3 (Fig 3). Similarly, ITS61.01/.02, ITS70.01, ITS104.01 and 5L7 did not neutralize SIV_mac_239 (Fig 5), but mediated potent ADCC against SIV_mac_239-infected cells (Fig 3). These results illustrate that neutralization and ADCC do not always correspond and reveal differences in some of the antibody-Env interactions that result in these responses.

To better understand the uncoupling of these antiviral functions, several of the antibodies that mediate ADCC without detectable neutralization were titrated for Env binding to the surface of SIV-infected cells. SIV_mac_239-infected cells were stained with ITS61.01, ITS70.01, ITS55 and 5L7 at concentrations ranging from 0.2 to 100 μg/ml. For comparison, these cells were also stained over the same range with ITS103.01 and with PGT145, which mediates ADCC against SIV-infected cells but does not neutralize SIV infectivity [67]. ITS103.01 exhibited 7-fold higher binding to Env than PGT145, as reflected by differences in area under the curve values. (S5 Fig). The level of Env staining for ITS61.01, ITS70.01, ITS55 and 5L7 was in the same range or lower than PGT145, indicating that these antibodies bind to Env with similar efficiency as PGT145 (S5 Fig). We previously demonstrated that the affinity of PGT145 for SIV Env is sufficient to trigger ADCC by cross-linking Env trimers on the surface of SIV-infected cells to FcɣR3A receptors on NK cells, but is not high enough to occupy enough Env trimers on virions to block SIV infectivity [67]. Thus, the uncoupling of ADCC from neutralization by ITS61.01, ITS70.01, ITS55 and 5L7 may be explained by the low affinity of these antibodies for Env in comparison to antibodies such as ITS103.01 that are capable of efficiently neutralizing virus infectivity.

**Fig 6:**
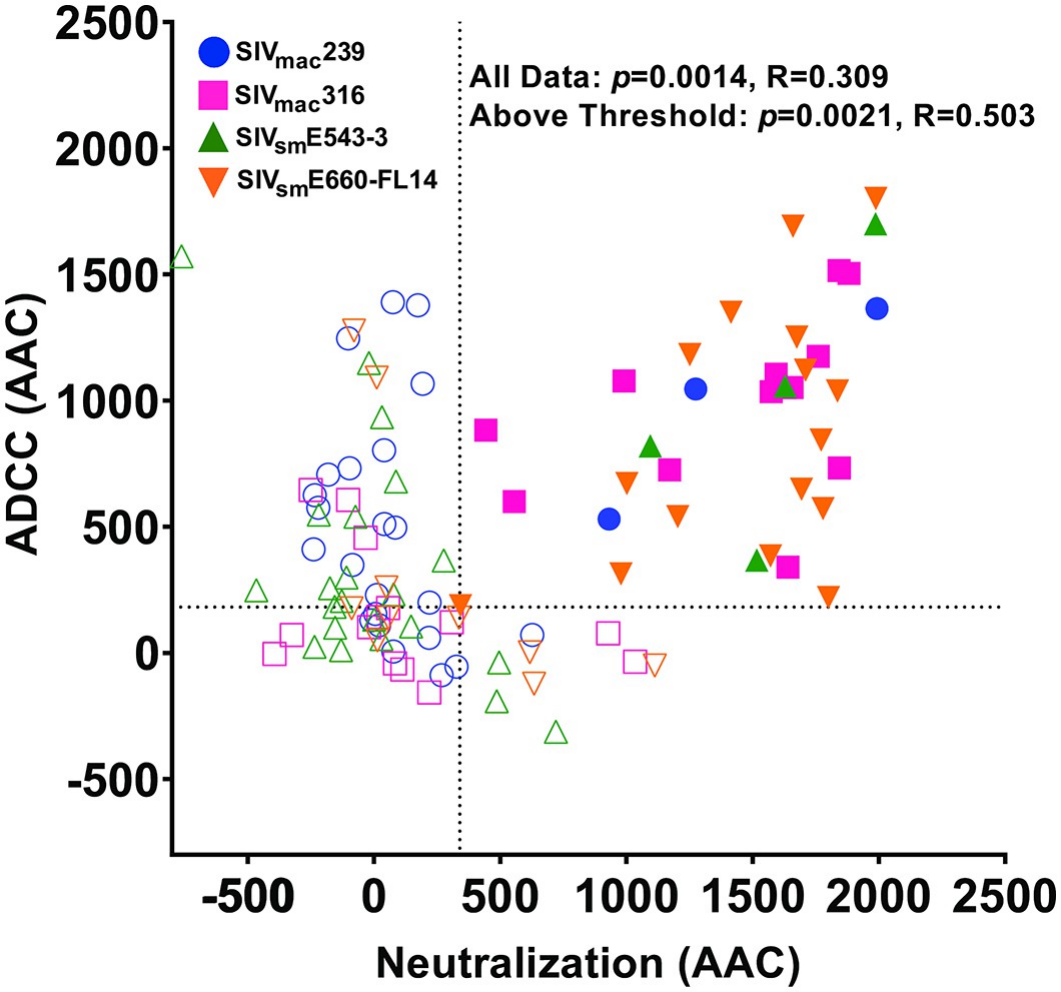
Comparison of ADCC and neutralization of viral infectivity. Area above the curve (AAC) values for ADCC and neutralization were calculated from the differences between the theoretical maximal luciferase induction and the reduction in luciferase activity at each antibody concentration. The dotted lines indicate thresholds for detectable ADCC and neutralization, which were set at one standard deviation above the absolute value of the mean negative ADCC and neutralization AAC values. Spearman’s rank order correlation coefficients were calculated for comparisons of ADCC and neutralization for all antibodies and for those antibodies that were above the thresholds for detectable responses.

Neutralization and ADCC were compared to investigate the relationship between these antiviral activities. As for ADCC, AAC values were calculated from neutralization curves to capture responses that did not reach an arbitrary threshold of neutralization and to provide a consistent metric for comparison with ADCC. Data for the ITS103 and ITS61 siblings was again averaged and treated as single antibodies to avoid biases from repeated measures of closely related antibodies. ADCC generally correlated with neutralization; however, this relationship was not especially strong (*p* = 0.0014, R = 0.309) (Fig 6). A closer inspection of the data suggested this correlation might be skewed by a large number of the antibodies which appear to lack one or both activities. Thresholds for detectable neutralization and ADCC were therefore established at one standard deviation above the absolute value of the mean of negative AAC values for neutralization and ADCC. Limiting the comparison to antibodies that were above these thresholds improved the strength but not the overall significance of the correlation (*p* = 0.0021, R = 0.503) (Fig 6). These analyses reveal a significant albeit imperfect correlation between ADCC and neutralization.

### Neutralization and ADCC activity of antibodies to CD4-inducible epitopes

Exposure of HIV-1 virions or infected cells to CD4 triggers Env to adopt an “open” CD4-bound conformation in which surfaces that are normally concealed in the “closed” pre-liganded conformation of the Env trimer become exposed to antibodies [68,69]. Antibodies to these surfaces, known as CD4-inducible (CD4i) epitopes, are thus dependent on CD4 for the ability to neutralize viral infectivity and to sensitize infected cells to ADCC. To determine the extent to which SIV-infected cells are susceptible to this class of antibodies, Env binding, ADCC, and neutralization were compared for two CD4i antibodies (ITS99 and 1.4H) in the presence and absence of soluble CD4 (sCD4).

**Fig 7:**
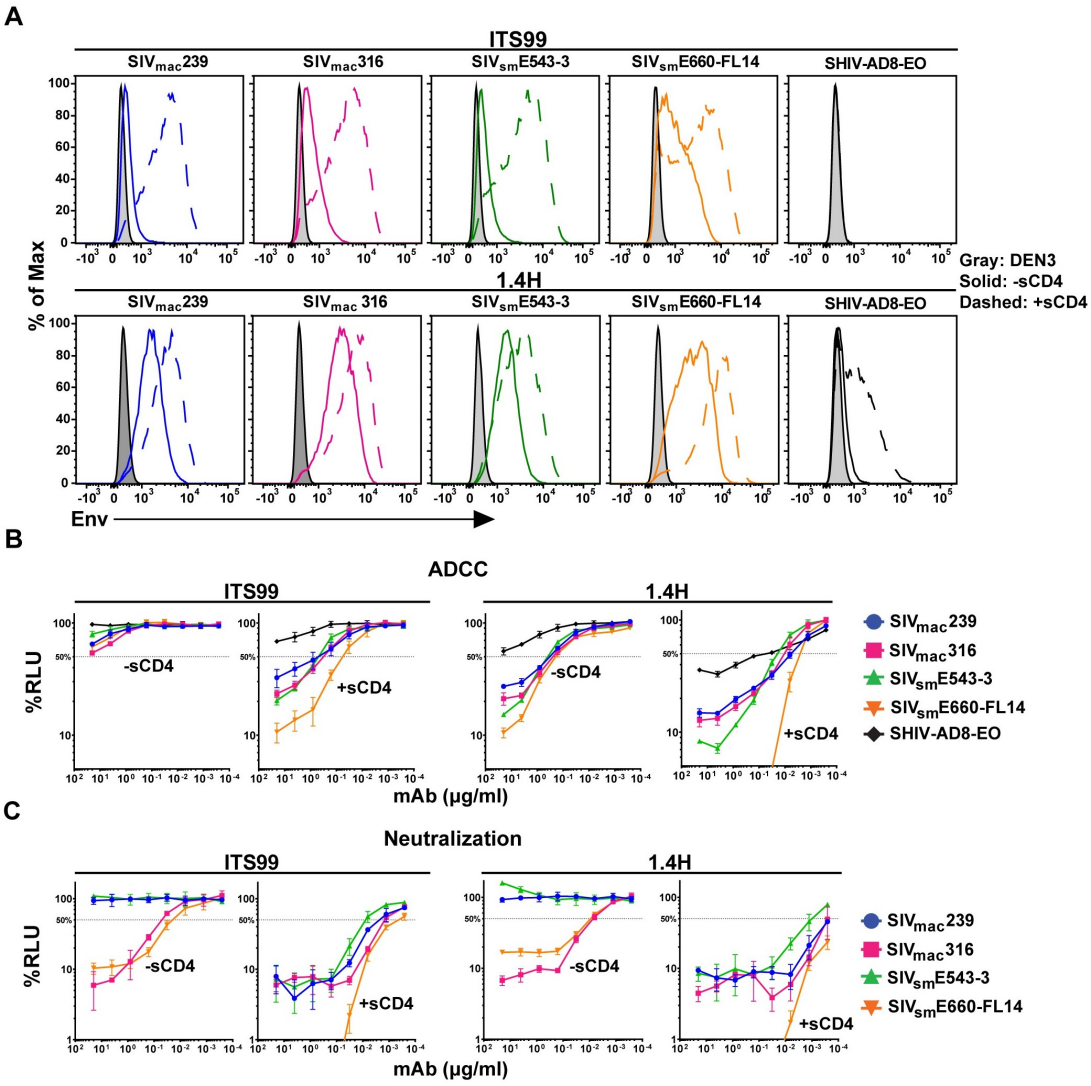
SIV Env binding, ADCC and neutralization by CD4-inducible antibodies. (A) Env staining on the surface of CEM.NKR-_CCR5_-sLTR-Luc cells infected with SIV_mac_239, SIV_mac_316, SIV_sm_E543-3, SIV_sm_E660-FL14 and SHIVAD8-EO was measured in the presence (dotted lines) and absence (solid lines) of soluble CD4 (sCD4) (10 μg/ml) relative to non-specific staining with DEN3 (shaded). Antibody binding to SIV-infected (Gag^+^ CD4_low_) cells was detected with an AF647-conjugated anti-human IgG F(ab′)_2_. (B) ADCC responses (% RLU) were measured as the dose-dependent loss of luciferase activity from SIV-infected CEM.NKR-_CCR5_-sLTR-Luc cells after an 8-hour incubation with a rhesus macaque CD16+ NK cell line at a 10:1 E:T ratio and the indicated concentrations of each antibody in the presence and absence of sCD4 (10 μg/ml). The plotted values represent the mean and standard deviation (error bars) for triplicate wells and the dotted line indicates half-maximal ADCC. (C) Neutralization of SIV_mac_239, SIV_mac_316, SIV_sm_E543-3 and SIV_sm_E660-FL14. Dilutions of SIV were prepared and incubated with and without sCD4 (10 μg/ml) and the indicated concentrations of each antibody for one hour before addition to TZM-bl cells. Three days post-infection, neutralization (% RLU) was calculated from the luciferase activity in cell lysates. The error bars indicate standard deviation of the mean for triplicate wells and the dotted line indicates 50% neutralization.

ITS99 and 1.4H bound to SIV-infected CEM.NKR-_CCR5_-sLTR-Luc cells and to primary rhesus macaque CD4+ T cells in the absence of sCD4. Env staining was markedly higher for 1.4H than for ITS99. Env staining was also higher for both antibodies on cells infected with neutralization-sensitive SIV_mac_316 and SIV_sm_E660-FL14 than with neutralization-resistant SIV_mac_239 or SIV_sm_E543-3 (Fig 7A and S1 and S2 Figs). Treatment with sCD4 dramatically increased the intensity of staining by both antibodies for all four SIVs, consistent with the binding of these antibodies to conserved surfaces of Env exposed upon CD4 engagement (Fig 7A and S1 and S2 Figs). Surprisingly, CD4-induced Env staining by 1.4H was even detectable on cells infected with SHIV-AD8-EO, indicating that this antibody binds to an epitope that is at least partially conserved in HIV-1 Env (Fig 7A).

The ADCC responses of ITS99 and 1.4H closely corresponded to differences in their ability to bind to Env on the surface of virus-infected cells. In the absence of sCD4, ITS99 mediated low but detectable ADCC, and 1.4H directed efficient killing of cells infected with all four SIVs (Fig 7B). The addition of sCD4 greatly increased the susceptibility of virus-infected cells to both antibodies as reflected by reductions in the antibody concentrations required for half-maximal killing (Fig 7B). Thus, CD4-induced conformational changes in Env significantly enhance the breadth and potency of ITS99- and 1.4H-mediated ADCC responses.

ITS99 and 1.4H were also tested for the ability to neutralize each of the SIVs in the presence and absence of sCD4. In the absence of sCD4, ITS99 and 1.4H efficiently neutralized SIV_mac_316 and SIV_sm_E660-FL14 but were unable to neutralize SIV_mac_239 or SIV_sm_E543-3, consistent with differences in the sensitivity of these viruses to antibodies (Fig 7C). However, similar to ADCC, treatment with sCD4 dramatically increased the susceptibility of all four SIVs to neutralization (Fig 7C). These results confirm CD4-induced changes in the sensitivity of SIV to neutralization by ITS99 and 1.4H and reveal parallels between neutralization and ADCC by CD4i antibodies.

Additional characterization of ITS104.01 revealed that this antibody also recognizes a CD4-inducible epitope. ITS104.01 mediates detectable ADCC against cells infected with SIV_mac_239 and SIV_sm_E543-3 but does not neutralize these viruses (Figs 3 and 5). Similar to ITS99, sCD4 increased Env staining and the sensitivity of SIV_mac_239-infected cells to ADCC in the presence of ITS104.01 (S6 Fig). Thus, limited Env binding and ADCC may occur in the absence of detectable neutralization for certain antibodies that target CD4-inducible epitopes.

### Soluble CD4 facilitates Env Binding and ADCC by the V2-specific antibodies NCI05 and NCI09

In light of evidence that CD4 binding induces conformational rearrangement of the V1/V2 loops [70–72], we postulated that differences in the binding of NCI05 and NCI09 to monomeric gp120 versus Env on the surface of SIV-infected cells may reflect CD4-inducible changes in V2. These antibodies were therefore tested for Env binding and ADCC against SIV-infected cells in the presence and absence of soluble CD4. With the exception of a low level of NCI05 staining on SIV_sm_E660-FL14-infected cells as noted above, NCI05 and NCI09 did not bind or mediate detectable ADCC without sCD4 (S7A Fig). However, treatment with sCD4 increased NCI05 binding to SIV_sm_E660-FL14-infected cells and rendered these cells susceptible to ADCC (S7A and S7B Fig). In the presence of sCD4, ADCC was also detectable against SIV_sm_E543-3-infected cells with NCI05 and against SIV_sm_E660-FL14-infected cells with NCI09 (S7B Fig). Soluble CD4 did not increase ADCC responses to SIV_mac_239- or SIV_mac_316-infected cells, indicating that sensitivity to sCD4 is strain-dependent. These findings nevertheless demonstrate that CD4 engagement can expose the V2 epitopes for NCI05 and NCI09, which may in turn explain the inability of these antibodies to bind to pre-liganded conformations of Env necessary for ADCC against SIV-infected cells in the absence of sCD4.

## Discussion

The isolation of monoclonal antibodies to the SIV envelope glycoprotein directly from SIV-infected or vaccinated rhesus macaques provides a valuable resource for investigating antibody-mediated protection in this species as a nonhuman primate model for HIV/AIDS [34,35]. In contrast to HIV-1 Env-specific antibodies, which require the use of recombinant SHIVs to assess protection, the protection afforded by these antibodies can be evaluated under more physiological conditions using SIV challenge strains that are well-adapted for replication in macaques. Rhesus macaque antibodies also retain natural interactions with macaque Fc receptors [51] and minimize ADA that can complicate repeated or sustained antibody delivery to animals [35]. Env expression levels for HIV-1 and SIV are very similar, reflecting conserved endocytosis motifs in the gp41 cytoplasmic domain and the incorporation of comparable numbers of Env trimers into virions [73–77]. With increasing interest in the development of vaccines and immunotherapies to take advantage of Fc-dependent effector functions such as ADCC, it is important to understand the capacity of Env-specific antibodies to mediate these activities and their relationship to virus neutralization. Using an assay specifically designed to measure the ability of antibodies to direct NK cell killing of virus-infected cells expressing physiologically relevant conformations of Env [63], we measured the ADCC responses of a diverse collection of Env-specific antibodies against cells infected with neutralization-resistant and neutralization-sensitive SIV isolates. ADCC responses were compared to Env binding on the surface of SIV-infected cells and to the neutralization of SIV infectivity.

Overall, we observed a good correlation between neutralization and ADCC. These results are consistent with previous analyses of HIV-1 Env-specific antibodies showing that most antibodies that are capable of binding to Env on virions to block infectivity are also capable of binding to Env on the surface of virus-infected cells to mediate ADCC [78–80]. The correspondence between these antiviral activities is best illustrated by ITS103, which exhibited the most potent neutralization and the highest ADCC responses of all of the antibodies tested. Nevertheless, this relationship was not absolute, as instances of ADCC in the absence of neutralization and neutralization in the absence of ADCC were also observed.

Several antibodies were found to mediate ADCC against SIV_mac_239- or SIV_sm_E543-3-infected cells without detectable neutralization of these viruses. This pattern was most common among the HMP-specific antibodies such as ITS54, ITS55, ITS61.01/02 and ITS70.01, but was also observed for 5L7 and a couple of the antibodies to the V1/V2 region (ITS03 and ITS06.02). Each of these antibodies exhibited ADCC responses to cells infected with SIV_mac_239 or SIV_sm_E543-3 as well as SIV_mac_316 and/or SIV_sm_E660-FL14, but they did not neutralize either SIV_mac_239 or SIV_sm_E543-3. Neutralization was detectable however against SIV_mac_316 and SIV_sm_E660-FL14, which suggests that the epitopes for these antibodies are present on both infected cells and virions but are inaccessible on neutralization-resistant viruses. One interpretation of these observations is that virus-infected cells express more heterogeneous conformations of Env than virions and that some of these conformations render infected cells more sensitive to antibodies. This appears to be the case for lab-adapted HIV-1 [78,81]. However, the primate lentiviruses have evolved molecular mechanisms to tightly control both the amount and the conformation of Env expressed on the cell surface [67,73,82], which greatly reduces the susceptibility of primary HIV-1 isolates to ADCC [78–80]. An alternative explanation may reflect differences in the binding affinity of antibodies required to mediate ADCC versus neutralization. This possibility was recently illustrated by the cross-reactivity of PGT145 with SIV Env [67]. PGT145 binds to a conserved epitope at the V2 apex of SIV Env in the same way that it binds to its HIV-1 Env epitope, but with lower affinity. Although the low affinity of PGT145 for SIV Env was sufficient to cross-link enough Env trimers on the surface of virus-infected cells with Fcɣ receptors on NK cells to trigger ADCC, it was not sufficient to bind enough Env trimers on virions to neutralize infectivity [67]. Similar to PGT145, we found that several other antibodies that mediate ADCC without detectable neutralization (ITS55, ITS61.01, ITS70.01 and 5L7) also bind to Env with lower affinity than an antibody that can efficiently neutralize virus infectivity. Thus, differences in the exposure of epitopes on virus-infected cells and virions and/or a lower binding affinity for Env could account for the ability of certain antibodies to mediate ADCC without detectable neutralization.

A few instances of neutralization in the absence of ADCC were also observed. These include the gp41 MPER antibodies ITS112.01 and ITS113.01 and the gp120 CD4bs antibody ITS01. In each case, these antibodies exhibited little if any Env staining, strongly suggesting that their failure to mediate ADCC reflects their inability to bind to Env on the surface of virus-infected cells. For ITS112.01 and ITS113.01, these results are consistent with exposure of the MPER epitope on a transient gp41 intermediate during the process of viral fusion that is not typically exposed on virus-infected cells [83,84], and with previous studies by our group and others showing that the HIV-1 MPER antibodies (2F5, 4E10 and 10E8) do not mediate ADCC against virus-infected cells [78,79]. The inability of ITS01 to bind or direct ADCC against cells infected with any of the SIV isolates is more difficult to understand in light of its neutralization of SIV_mac_316 and SIV_sm_E660-FL14. It is possible that exposure of the epitope for this antibody is dependent on conformational changes in Env that are completed after the release of infectious virus particles. Alternatively, this antibody may bind to Env in an orientation that is sterically hindered on the surface of infected cells, but not on virions. In this regard it is perhaps relevant that unlike more potent antibodies to the CD4bs such as ITS103 and IST102.03, ITS01 only blocks infection by neutralization-sensitive viruses. A more precise molecular explanation for the uncoupling of neutralization and ADCC by ITS01 may require further biophysical or structural characterization of antibody-Env complexes.

CD4 engagement induces Env trimers to transition from a “closed” pre-liganded conformation to an “open” CD4-bound state that is susceptible to recognition by CD4-inducible antibodies. CD4i-antibodies are abundant in most HIV-1 infected individuals as a consequence of B cell responses to viral debris, such as monomeric gp120. These antibodies are considered non-neutralizing since primary HIV-1 isolates are highly resistant to CD4i antibodies [78,85,86]. However, treatment with soluble CD4 or CD4-mimetic compounds can sensitize cell-free HIV-1 and virus-infected cells to CD4i-antibodies [68,85,87]. Cells infected with *vpu*- or *nef*-deficient HIV-1 strains that are impaired for CD4 downmodulation are also susceptible to ADCC as a result of the formation of CD4-Env complexes on the cell surface that expose CD4i epitopes [68,85,88]. These properties have led to a great deal of confusion regarding the importance of CD4i antibodies, since ADCC assays that use CD4^+^ target cells coated with recombinant gp120/gp140 [89,90], target cells infected with *vpu*- or *nef*-deficient viruses [91], or that do not differentiate HIV-infected cells from uninfected bystander cells [60,92,93] greatly overestimate the contribution of CD4i-antibodies to ADCC responses [94].

Two antibodies targeting CD4i epitopes of SIV Env (ITS99 and 1.4H) were evaluated for Env binding, ADCC and neutralization in the presence and absence of soluble CD4. Similar to HIV-1 CD4i-antibodies, ITS99 and 1.4H failed to block the infectivity of neutralization-resistant primary SIV isolates (SIV_mac_239 and SIV_sm_E543-3) in the absence of sCD4. Although Env binding and ADCC were detectable without sCD4, the addition of sCD4 potently enhanced ADCC and neutralization by both antibodies. CD4-induced increases in sensitivity to ADCC corresponded to increases in Env binding. Increases in susceptibility to neutralization also reflected increases in Env binding and ADCC. These results are consistent with CD4i responses to HIV-1 and reinforce the general correlation between neutralization and ADCC.

NCI05 and NCI09 bind to non-overlapping epitopes in V2 and the specificity of these antibodies has been associated with a decreased risk of mucosal SIV transmission in vaccinated macaques [21,95,96]. We confirmed that NCI09 and NCI05 bind to gp120-coated cells in accordance with their previously reported ADCC activity against gp120-coated target cells [21]. However, we were unable to detect Env binding or ADCC with these antibodies in assays using SIV-infected cells. These discrepancies likely reflect conformational differences in the exposure of the V2 epitopes for NCI05 and NCI09 on monomeric gp120 versus Env trimers on SIV-infected cells. In support of this, we found that treatment with soluble CD4 increased antibody binding and rendered SIV-infected susceptible to ADCC. These results are consistent with studies showing that CD4 binding to HIV-1 Env induces conformational rearrangements of the V1/V2 loops [70–72] and with similar CD4-dependent ADCC activity of non-neutralizing antibodies to the inner domain and co-receptor binding sites of HIV-1 gp120 [68,94]. Structural features of Env that confer resistance to neutralizing antibodies [3] also protect virus-infected cells from ADCC [78–80,97]. Thus, antibodies to surfaces of gp120 that are normally concealed in Env trimers and are unable to mediate ADCC against virus-infected cells may still bind to monomeric gp120 and direct ADCC in assays using gp120-coated cells [94].

The present study reveals fundamental insights into the relationship between ADCC, Env binding, and neutralization for a diverse collection of SIV Env-specific antibodies. Neutralizing antibodies to the CD4bs and non-neutralizing antibodies to CD4i epitopes were identified with especially broad and potent ADCC against genetically distinct SIVs. The overall correlation between ADCC and neutralization implies, perhaps not surprisingly, that most antibodies that are capable of binding to functional Env trimers on virions to block infectivity are also capable of binding to Env on virus-infected cells to mediate ADCC. Nevertheless, several antibodies were found to mediate ADCC without detectable neutralization, or neutralization without detectable ADCC, pointing to key differences in the way that certain antibodies interact with Env on virus-infected cells and virions that can uncouple these activities. These findings provide a valuable dataset and conceptual framework for investigating antiviral effector functions of antibodies in macaques as a preclinical model for HIV/AIDS.

## Materials and methods

### Virus production

Virus stocks were produced by transfection of HEK-293T cells with infectious molecular clones (IMCs) for SIV_mac_239, SIV_mac_316 TM-open, SIV_sm_E543-3 and SIV_sm_E660-FL14 [38,39,57–59]. Deletions were introduced into the *vif* genes of SIV_mac_239 (nucleotide 155), SIV_mac_316 TM-open (nucleotide 155) and SIV_sm_E543-3 (nucleotides 108–109) that result in frameshift mutations followed by multiple stop codons to prevent spreading infection of vesicular stomatitis virus glycoprotein (VSV G) pseudotyped viruses. HEK-293T cells were transfected with *vif*-deleted SIV_mac_239, SIV_mac_316 and SIV_sm_E543-3 IMCs together with the VSV G expression construct pHDM.NJ, or with wild-type SIV_sm_E660-FL14 without pHDM.NJ, using GenJet version II transfection reagent (SignaGen). Cell culture supernatants were collected 48 hours post-transfection and concentrated in 50kD centrifugal filters (EMD Millipore) before determining SIV p27 capsid concentrations by antigen-capture ELISA (ABL Inc.). Aliquots of concentrated virus stocks were stored at -80°C.

### Antibodies

SIV Env-specific antibodies were isolated and produced as previously described [34,35]. Briefly, immunoglobulin variable region gene sequences were determined from SIV Env-specific B cells which were individually sorted by flow cytometry using a fluorescently labelled SIV gp140 competitive probe binding strategy and 1JO8 scaffolded SIV_sm_E660 V1/V2 probes. These sequences were cloned into rhesus Igɣ, IgLκ and IgLλ expression vectors. Full-length IgG (rhesus IgG1) was expressed by co-transfection of heavy and light chain plasmids into 293Freestyle cells and purified using Protein A Sepharose beads (GE Healthcare) according to manufacturer’s instructions. 5L7 was expressed in Expi293 cells from a recombinant AAV vector very similar to the ‘ssAAV (H+L)’ plasmid [25] with the following exceptions: the signal peptide on both IgG chains corresponded to the sequence from human VH4 (UniProt entry O95973), the skip peptide separating heavy and light chains was P2A instead of F2A, and the heavy chain of 5L7 was C-terminally tagged with the rhodopsin-derived peptide corresponding to the epitope for 1D4 mouse monoclonal antibody (TETSQVAPA). The plasmid backbone was a gift from Dr. Matthew Gardner, Emory University. 5L7 was purified from cell culture supernatant on rProtein A GraviTrap columns (GE Healthcare) according to the manufacturer’s recommendations, except that the bound antibody was eluted with 3 ml of 25 mM sodium phosphate/citrate buffer pH 3.0 into a 0.2 ml of 1.0 M sodium carbonate buffer pH 9.3.1.4H antibody sequences were originally isolated from Epstein-Barr virus transformed B cells from an HIV-2 infected patient as previously described [53,54,98]. Plasmids encoding 1.4H human IgG1 heavy and light chain sequences were generously provided by James Robinson (Tulane University). 1.4H antibody was produced by co-transfection of heavy and light chain plasmids into Expi293 cells and purified from culture supernatant using rProtein A GraviTrap columns according to manufacturer’s instructions.

### Envelope staining on the surface of infected cells

CEM.NKR-_CCR5_-sLTR-Luc cells were infected with *vif*-deleted SIV_mac_239, SIV_mac_316 and SIV_sm_E543-3 pseudotyped with VSV G, or with wild-type SIV_sm_E660-FL14, in the presence of 40 μg/ml Polybrene and centrifuged for 1 hour at 1200 g. Antibody binding to Env was assessed by staining the cells 3–5 days post-infection. Cells were treated with a Live/Dead NEAR IR viability dye (Invitrogen), washed in PBS containing 1% FBS (staining buffer), and stained on ice for 30 minutes with 10 μg/ml of Env-specific antibody or with a dengue virus-specific antibody (DEN3) as a negative control. For soluble CD4 (sCD4) experiments, cells were stained on ice for 30 minutes with 10 μg/ml of anti-SIV Env antibody and 10 μg/ml sCD4-183 (NIH AIDS Reagent Program). Cells were then washed and stained on ice with an AF647-conjugated goat anti-human F(ab′)_2_ (Jackson Immunoresearch), followed by PE-Cy7-conjugated anti-CD4 (Clone OKT4, Biolegend). For intracellular p27 staining, cells were fixed in PBS with 2% paraformaldehyde, washed in staining buffer and stained with FITC-conjugated anti-SIV Gag antibody (clone 552F12) in Perm/Fix Medium B (Invitrogen). Cells were washed, fixed in PBS with 2% paraformaldehyde and analyzed on a BD LSRII flow cytometer. Antibody binding to Env was assessed by Env staining on the surface of SIV-infected (CD4_low_Gag^+^) cells.

Primary rhesus macaque CD4+ T cells were infected with SIV and stained for surface expression of Env. Peripheral blood mononuclear cells (PBMCs) were isolated from whole blood on Ficoll-Paque PLUS gradients. CD8^+^ T cells and NK cells were depleted using 0.7 μg/ml mouse anti-CD8 antibody (clone SK11), followed by sheep anti-mouse magnetic beads (Dynabeads) [99]. After CD8 depletion, cells were activated with 5 μg/ml concavalin A for 3 days, washed, and cultured for 3 days in medium with 20 U/ml IL-2 (R&D Systems). Tissue culture-treated flasks were laid on their side to deplete adherent cells such as monocytes and macrophages [100]. CD4+ T cells were then infected with SIV by spinoculation at 1200 g for 2 hours in the presence of Polybrene (40 μg/ml): SIV_mac_239 (500 ng p27/10^6^ cells), SIV_mac_316 (375 ng p27/10^6^ cells), SIV_sm_E543-3 (500 ng/10^6^ cells) and SIV_sm_E660-FL14 (340 ng/10^6^ cells). Five days post-infection, cells were stained with a viability dye, followed by incubation with 10 μg/ml anti-SIV Env antibody (with or without 10 μg/ml sCD4-183) for 30 minutes on ice and an AF647-conjugated goat anti-human F(ab′)_2_ as described above. Cells were then surface stained on ice with PE-Cy7-conjugated anti-CD4 and BV421-conjugated anti-CD8 (Clone SK11, Biolegend). For intracellular p27 staining, cells were fixed in PBS plus 2% paraformaldehyde, washed and stained with AF488-conjugated anti-SIV Gag antibody (clone 552F12 for SIV_mac_ strains) or with AF488-conjugated anti-HIV Gag antibody (clone 183-H2 for SIV_sm_ strains) in Perm/Fix Medium B. Cells were then washed, fixed in PBS with 2% paraformaldehyde before analysis on a BD LSRII flow cytometer. Antibody binding to Env was assessed by Env staining on the surface of SIV-infected (CD4_low_Gag^+^) cells.

Antibodies NCI05 and NCI09 were tested for binding to cells coated with monomeric SIV gp120. CEM.NKR-_CCR5_-sLTR-Luc cells were incubated with 50 μg/ml of SIV_mac_251-M766 gp120, SIV_mac_251-M766 ΔV1 gp120, or SIV_mac_239 gp120 for 2 hours at 37°C. SIV_mac_251-M766 ΔV1 gp120 contains a 45 amino acid deletion in the V1 region (Env residues 119–163) which was shown to retain an α-helical conformation of V2 [21]. Coated cells were washed and treated with a Live/Dead NEAR IR viability dye before staining with 10 μg/ml of the Env-specific antibodies, NCI05 or NCI09. Cells were then washed and stained on ice with an AF647-conjugated goat anti-human F(ab′)_2_ followed by PE-Cy7-conjugated anti-CD4 as described above. Uncoated cells and non-specific DEN3 antibody were used as negative controls. Antibody binding to monomeric gp120 was assessed by staining on the surface of live CD4^+^ gp120 coated cells.

### ADCC assay

ADCC was measured as described previously described [63,94]. CEM.NKR-_CCR5_-sLTR-Luc cells were infected with VSV G-trans-complemented, *vif*-deleted SIV_mac_239, SIV_mac_316 or SIV_sm_E543-3, or with wild-type SIV_sm_E660-FL14, in the presence of 40 μg/ml Polybrene and centrifuged for 1 hour at 1200 g. After 2–4 days of infection, the CEM.NKR-_CCR5_-sLTR-Luc cells were incubated for 8 hours with an NK cell line (KHYG-1 cells) transduced with rhesus macaque CD16 at a 10:1 effector to target (E:T) ratio in the presence of antibodies. CD16+ KHYG-1 cells (10^5^ cells/well) were incubated with CEM.NKR-_CCR5_-sLTR-Luc cells (10^4^ cells/well) in triplicate wells of 96-well plates (0.2 ml/well). For antibodies to CD4-inducible epitopes, the assay was performed with or without 10 μg/ml sCD4-183 (NIH AIDS Reagent Program). ADCC was determined from the dose-dependent loss of luciferase activity. ADCC responses were calculated as the remaining luciferase activity (% RLU) by dividing the difference in RLU between SIV-infected cells in the presence of antibody and uninfected cells without antibody (experimental—background) by the difference in RLU between SIV-infected cells and uninfected cells in the absence antibody (maximal–background) and multiplying by 100.

### Neutralization assay

Virus neutralization was measured using a standard TZM-bl reporter assay as previously described [65,66]. Serial dilutions of antibodies were incubated for 1 hour at 37°C with viral supernatants at the following SIV p27 concentrations: 2 ng/well SIV_mac_239, 20 ng/well SIV_mac_316, 15 ng/well SIV_sm_E543-3, or 6 ng/well SIV_sm_E660-FL14. For antibodies to CD4-inducible epitopes, the assay was performed with or without 10 μg/ml sCD4-183. TZM-bl cells (10^4^ cells/well) were added after a 1 hour incubation and luciferase activity was measured after a 3-day incubation at 37°C. Virus neutralization was calculated as the dose-dependent inhibition of luciferase induction (RLU) by dividing the difference in RLU between wells containing virus in the presence of antibody and uninfected cells in the absence of antibody (experimental—background) by the difference in RLU between SIV-infected cells and uninfected cells in the absence of antibody (maximal–background) and multiplying by 100.

### Statistical analyses

All statistical analyses were performed using GraphPad Prism v.9.2. Geometric mean fluorescence intensity (gMFI) values for antibody binding to infected cells were calculated using FlowJo Ver 10.5.3 (Tree Star, Inc.). For comparison of Env binding, a gMFI ratio was calculated by dividing the gMFI of Env staining by the gMFI of staining with the DEN3 control antibody. To avoid biasing the correlations with negative data points, gMFI ratios with values less than one were interpreted as noise and used to define an arbitrary threshold of detection. This threshold of detectable Env binding was calculated as one standard deviation above the mean of gMFI ratios less than one. Similarly, negative area above the curve (AAC) values for ADCC and neutralization assays were interpreted as noise and used to define the arbitrary thresholds for detectable responses as one standard deviation above the absolute value of the mean of negative values. For all correlations, gMFI ratios and AAC values for ITS103 and ITS61 siblings were averaged and plotted as a single point to avoid biasing results from repeated measures of closely related antibodies. Relationships were evaluated using a Spearman’s rank order correlation. Correlations were performed both with all data points and with only data points above threshold values for comparison. A complete data set of the gMFI ratios for antibody binding to SIV-infected target cells (CEM.NKR-_CCR5_-sLTR-Luc cells) and primary CD4+ T cells and the AAC values for ADCC and neutralization used for these analyses is provided (S1 File).

## Supporting information

S1 Fig Env staining on the surface of SIV_mac_-infected rhesus macaque CD4+ T cells. Rhesus macaque PBMCs were CD8-depleted, activated with concanavalin A (5 μg/ml) and CD4+ T cells were expanded in medium with IL-2 (20 U/ml). Activated CD4+ T cells were infected with (A) SIV_mac_239 (blue) or (B) SIV_mac_316 (magenta). After 3–5 days, the cells were stained with each of the SIV Env-specific antibodies and with DEN3 as a control. Antibody binding to Env was detected by staining with AF647-conjugated anti-human IgG F(ab′)_2_. The lymphocytes were also stained for surface expression of CD4 and CD8, intracellular expression of the SIV Gag protein and for cell viability. The histograms depict Env staining (color) relative to non-specific DEN3 staining (shaded) on virus-infected (Gag^+^ CD4_low_) cells.
(TIF)

S2 Fig Env staining on the surface of SIV_sm_-infected rhesus macaque CD4+ T cells. Rhesus macaque PBMCs were CD8-depleted, activated with concanavalin A (5 μg/ml) and CD4+ T cells were expanded in medium with IL-2 (20 U/ml). Activated CD4+ T cells were infected with (A) SIV_sm_E543-3 (green) or (B) SIV_sm_E660-FL14 (orange). After 3–5 days, the cells were stained with each of the SIV Env-specific antibodies and with DEN3 as a control. Antibody binding to Env was detected by staining with AF647-conjugated anti-human IgG F(ab′)_2_. The lymphocytes were also stained for surface expression of CD4 and CD8, intracellular expression of the SIV Gag protein and for cell viability. The histograms depict Env staining (color) relative to non-specific DEN3 staining (shaded) on virus-infected (Gag^+^ CD4_low_) cells.
(TIF)

S3 Fig Gating strategy for antibody binding to Env on virus-infected cells. Representative gating is shown for ITS103.01 and DEN3 binding to SIV_mac_239-infected CEM.NKR-_CCR5_-sLTR-Luc cells (**A**) and primary rhesus macaque CD4^+^ T cells (**B**). Binding to Env was measured as the AF647 gMFI after gating on singlet, live, CD8^-^, infected (Gag^+^CD4_low_) cells.
(TIF)

S4 Fig V2-specific antibodies NCI05 and NCI09 bind to gp120-coated cells. CEM.NKR-_CCR5_-sLTR-Luc cells were coated with monomeric SIV_mac_251-M766 gp120, SIV_mac_251-M766 ΔV1 gp120, or SIV_mac_239 gp120 [21]. Coated cells were stained with the SIV gp120-specific antibodies NCI05 and NCI09 and the control antibody DEN3, followed by AF647-conjugated anti-human IgG F(ab′)_2_. The histograms plots depict Env staining in comparison to non-specific staining with DEN3 (shaded).
(TIF)

S5 Fig ADCC in the absence of neutralization reflects less efficient Env binding. SIV_mac_239-infected cells were stained over a 4-log range of antibody concentrations (0.2–100 μg/ml) to compare the efficiency of Env binding for ITS55, ITS61.01, ITS70.01 and 5L7 with the potent neutralizing antibody ITS103.01 (red) and with PGT145 (open circles), which mediates ADCC against SIV-infected cells but does not neutralize SIV infectivity. The dotted line indicates non-specific staining with DEN3 (100 ug/ml).
(TIF)

S6 Fig Soluble CD4 increases Env binding and ADCC for ITS104.01. (A) SIV_mac_239-infected cells were stained with 1.4H and ITS104.01 in the presence (open symbols) and absence (closed symbols) of soluble CD4 (sCD4) (10 μg/ml) over the indicated range of antibody concentrations. The dotted line indicates non-specific staining with DEN3 (100 ug/ml). (B) ADCC responses (% RLU) were measured as the dose-dependent loss of luciferase activity from SIV_mac_239-infected CEM.NKR-_CCR5_-sLTR-Luc cells after an 8-hour incubation with a rhesus macaque CD16^+^ NK cell line at a 10:1 E:T ratio and the indicated concentrations of ITS104.01 in the presence and absence of sCD4 (10 μg/ml). The plotted values represent the mean and standard deviation (error bars) for triplicate wells and the dotted line indicates half-maximal ADCC.
(TIF)

S7 Fig Soluble CD4 facilitates Env binding and ADCC for the V2-specific antibodies NCI05 and NCI09. (A) Env staining on the surface of CEM.NKR-_CCR5_-sLTR-Luc cells infected with SIV_mac_239, SIV_mac_316, SIV_sm_E543-3, SIV_sm_E660-FL14 and SHIV AD8-EO was measured in the presence and absence of soluble CD4 (sCD4) (10 μg/ml) relative to non-specific staining of SHIV AD8-EO-infected cells (shaded). Antibody binding to SIV-infected (Gag^+^ CD4_low_) cells was detected with an AF647-conjugated anti-human IgG F(ab′)_2_. (B) ADCC responses (% RLU) were measured as the dose-dependent loss of luciferase activity from SIV-infected CEM.NKR-_CCR5_-sLTR-Luc cells after an 8-hour incubation with a rhesus macaque CD16^+^ NK cell line at a 10:1 E:T ratio and the indicated concentrations of each antibody in the presence and absence of sCD4 (10 μg/ml). The plotted values represent the mean and standard deviation (error bars) for triplicate wells and the dotted line indicates half-maximal ADCC.
(TIF)

S1 File Area above the curve and gMFI ratio values for ADCC, neutralization, and binding to SIV-infected cells by SIV Env-specific antibodies. (XLS)
