# Supplementary figures and images for: Antibody-dependent cellular cytotoxicity, infected cell binding and neutralization by antibodies to the SIV envelope glycoprotein
Source: PLoS Pathog. 2023 May 30;19(5):e1011407. doi: 10.1371/journal.ppat.1011407 (PMC10256149; doi:10.1371/journal.ppat.1011407)

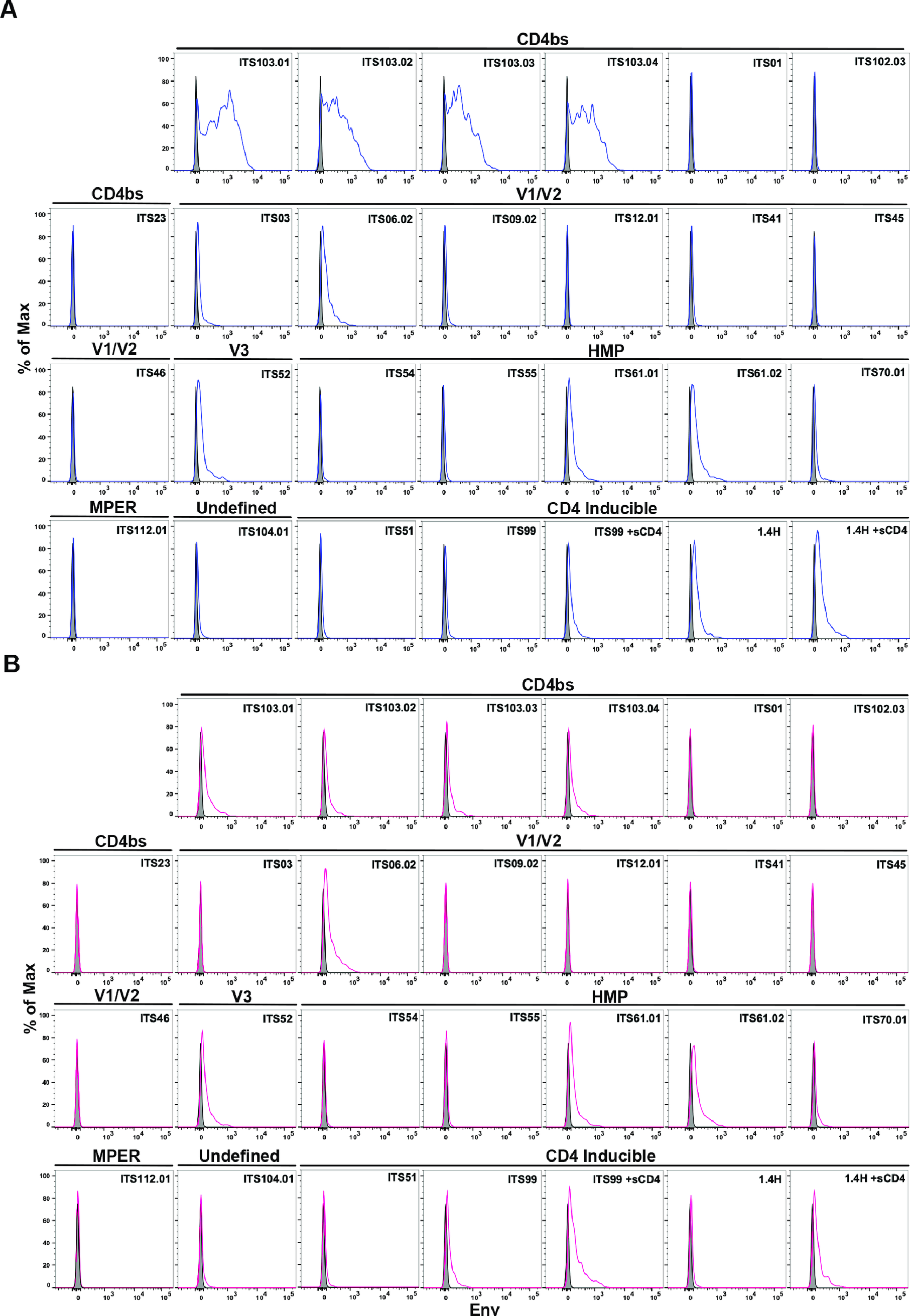

Supplement: S1 Fig — Rhesus macaque PBMCs were CD8-depleted, activated with concanavalin A (5 μg/ml) and CD4+ T cells were expanded in medium with IL-2 (20 U/ml). Activated CD4+ T cells were infected with (A) SIVmac239 (blue) or (B) SIVmac316 (magenta). After 3–5 days, the cells were stained with each of the SIV Env-specific antibodies and with DEN3 as a control. Antibody binding to Env was detected by staining with AF647-conjugated anti-human IgG F(ab′)2. The lymphocytes were also stained for surface expression of CD4 and CD8, intracellular expression of the SIV Gag protein and for cell viability. The histograms depict Env staining (color) relative to non-specific DEN3 staining (shaded) on virus-infected (Gag+ CD4low) cells. (TIF) [file ppat.1011407.s001.tif]

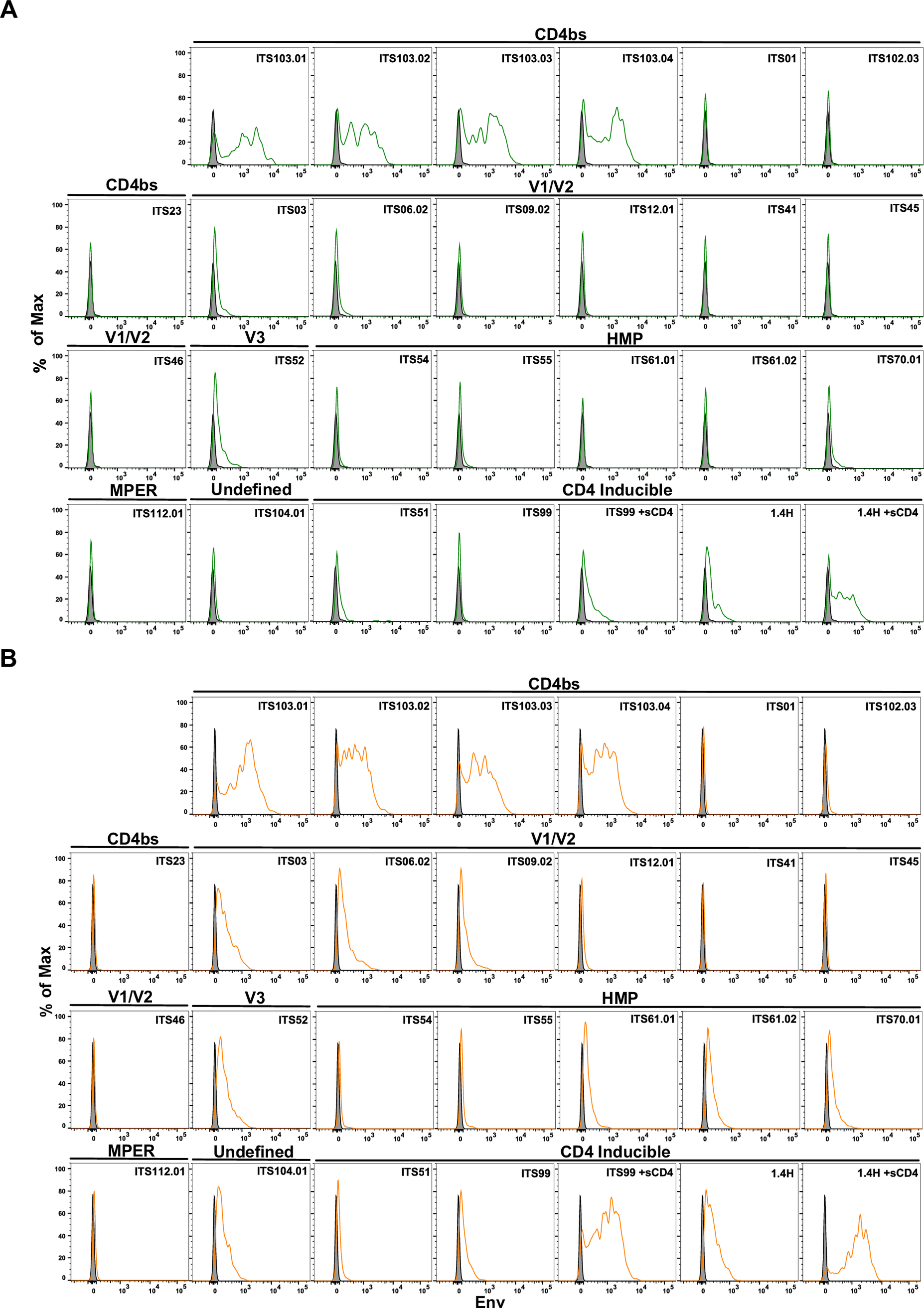

Supplement: S2 Fig — Rhesus macaque PBMCs were CD8-depleted, activated with concanavalin A (5 μg/ml) and CD4+ T cells were expanded in medium with IL-2 (20 U/ml). Activated CD4+ T cells were infected with (A) SIVsmE543-3 (green) or (B) SIVsmE660-FL14 (orange). After 3–5 days, the cells were stained with each of the SIV Env-specific antibodies and with DEN3 as a control. Antibody binding to Env was detected by staining with AF647-conjugated anti-human IgG F(ab′)2. The lymphocytes were also stained for surface expression of CD4 and CD8, intracellular expression of the SIV Gag protein and for cell viability. The histograms depict Env staining (color) relative to non-specific DEN3 staining (shaded) on virus-infected (Gag+ CD4low) cells. (TIF) [file ppat.1011407.s002.tif]

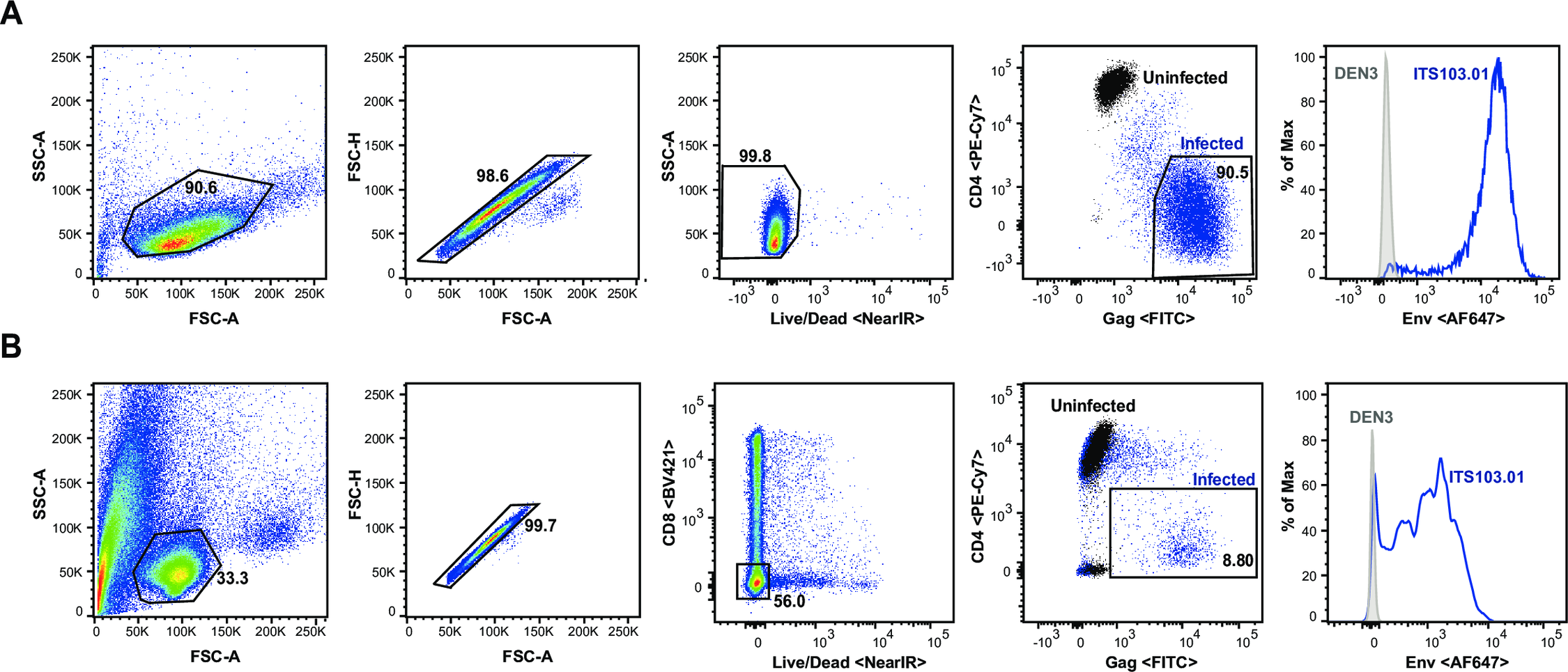

Supplement: S3 Fig — Representative gating is shown for ITS103.01 and DEN3 binding to SIVmac239-infected CEM.NKR-CCR5-sLTR-Luc cells (A) and primary rhesus macaque CD4+ T cells (B). Binding to Env was measured as the AF647 gMFI after gating on singlet, live, CD8-, infected (Gag+CD4low) cells. (TIF) [file ppat.1011407.s003.tif]

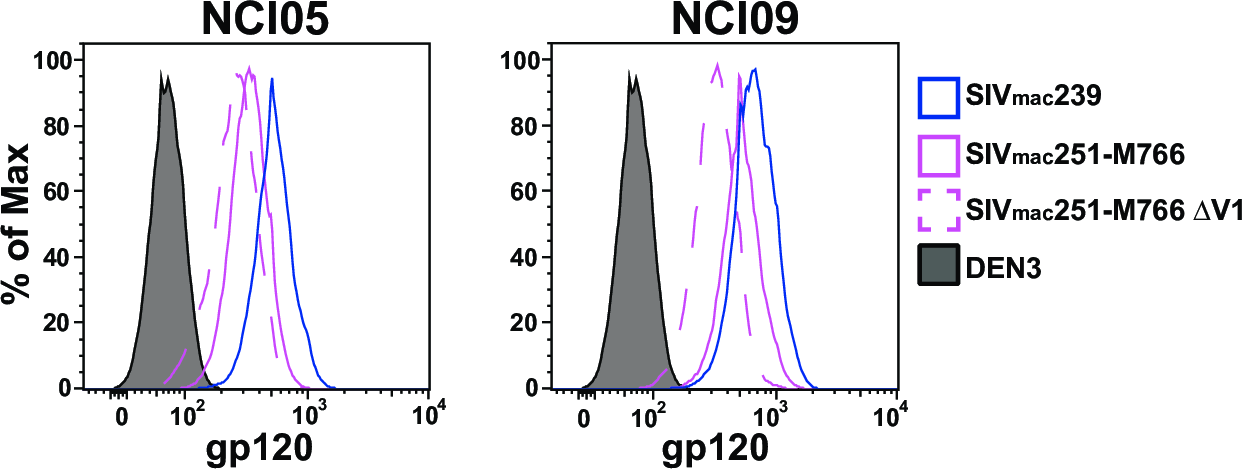

Supplement: S4 Fig — CEM.NKR-CCR5-sLTR-Luc cells were coated with monomeric SIVmac251-M766 gp120, SIVmac251-M766 ΔV1 gp120, or SIVmac239 gp120 [21]. Coated cells were stained with the SIV gp120-specific antibodies NCI05 and NCI09 and the control antibody DEN3, followed by AF647-conjugated anti-human IgG F(ab′)2. The histograms plots depict Env staining in comparison to non-specific staining with DEN3 (shaded). (TIF) [file ppat.1011407.s004.tif]

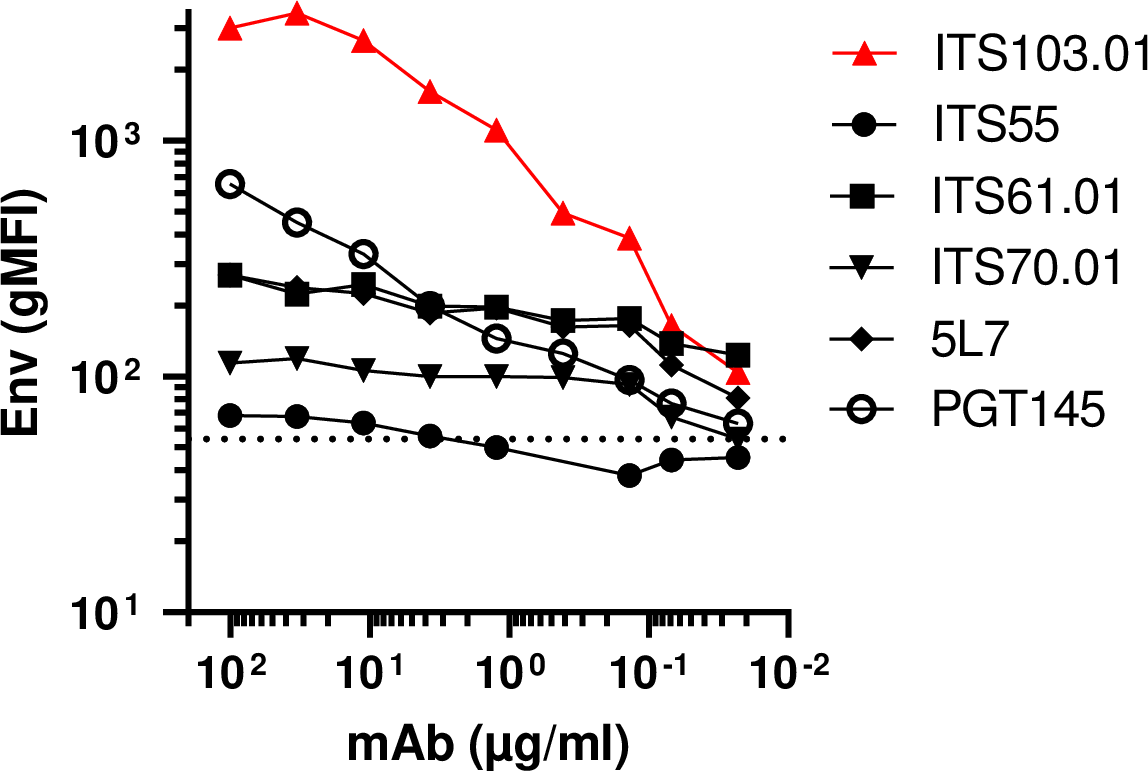

Supplement: S5 Fig — SIVmac239-infected cells were stained over a 4-log range of antibody concentrations (0.2–100 μg/ml) to compare the efficiency of Env binding for ITS55, ITS61.01, ITS70.01 and 5L7 with the potent neutralizing antibody ITS103.01 (red) and with PGT145 (open circles), which mediates ADCC against SIV-infected cells but does not neutralize SIV infectivity. The dotted line indicates non-specific staining with DEN3 (100 ug/ml). (TIF) [file ppat.1011407.s005.tif]

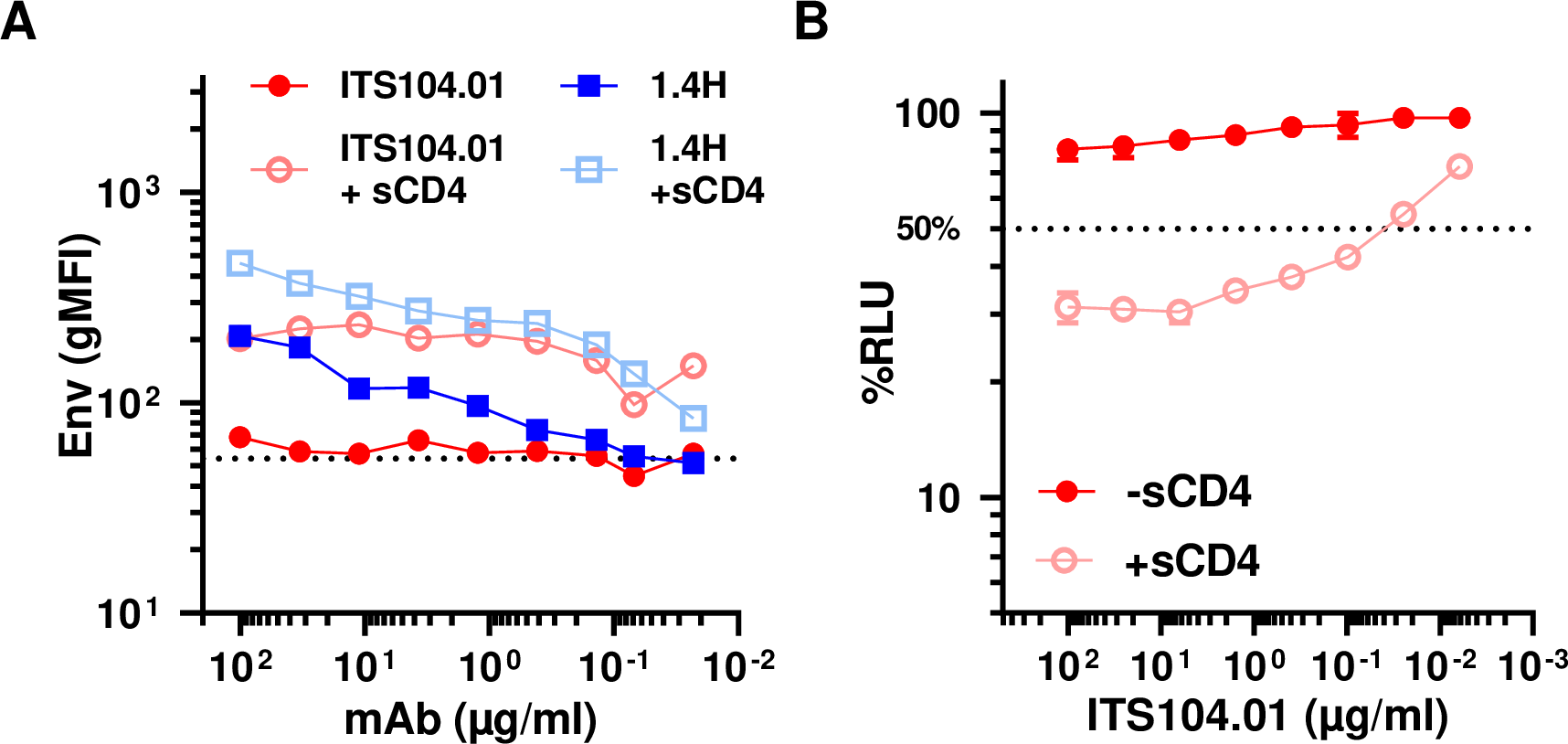

Supplement: S6 Fig — (A) SIVmac239-infected cells were stained with 1.4H and ITS104.01 in the presence (open symbols) and absence (closed symbols) of soluble CD4 (sCD4) (10 μg/ml) over the indicated range of antibody concentrations. The dotted line indicates non-specific staining with DEN3 (100 ug/ml). (B) ADCC responses (% RLU) were measured as the dose-dependent loss of luciferase activity from SIVmac239-infected CEM.NKR-CCR5-sLTR-Luc cells after an 8-hour incubation with a rhesus macaque CD16+ NK cell line at a 10:1 E:T ratio and the indicated concentrations of ITS104.01 in the presence and absence of sCD4 (10 μg/ml). The plotted values represent the mean and standard deviation (error bars) for triplicate wells and the dotted line indicates half-maximal ADCC. (TIF) [file ppat.1011407.s006.tif]

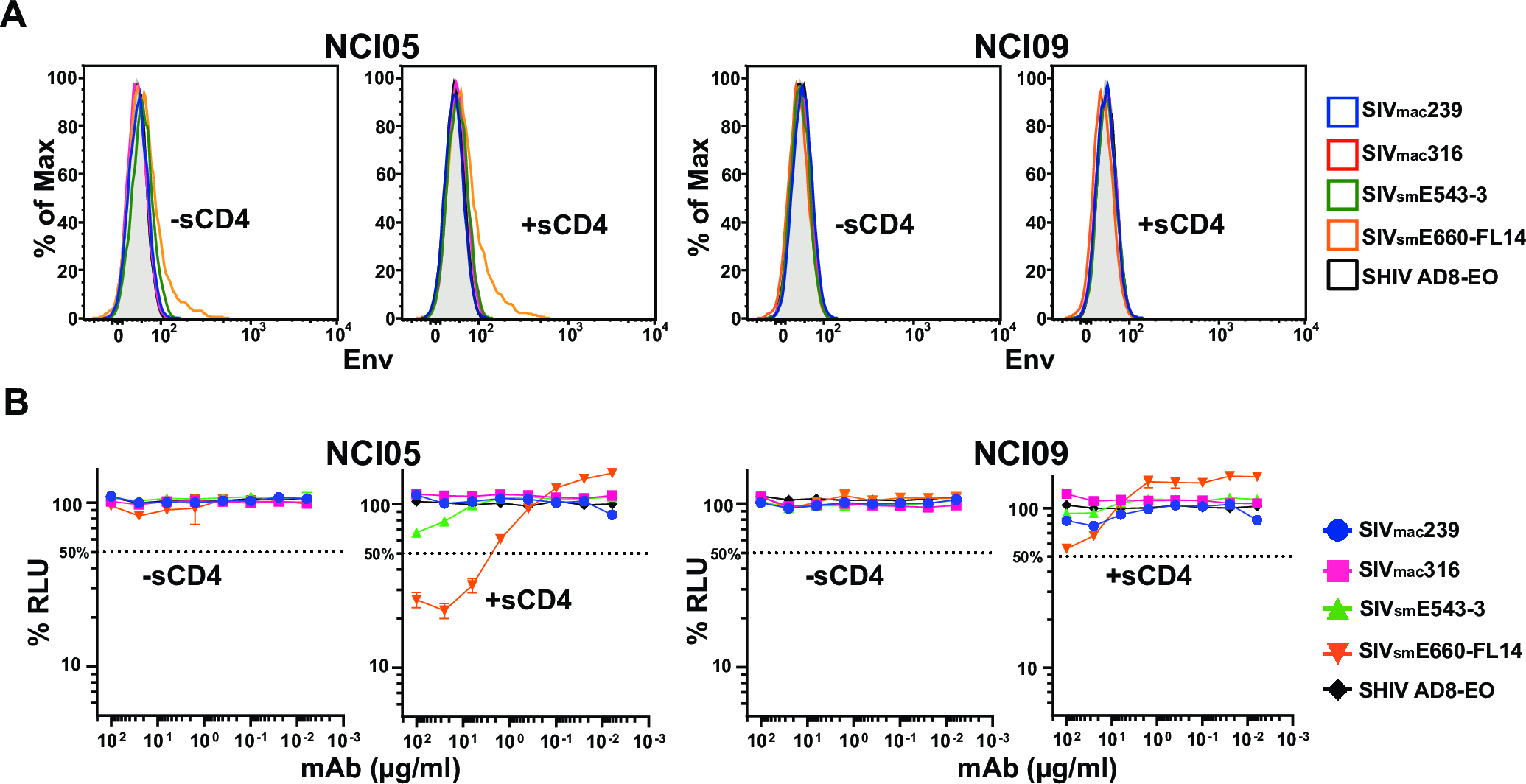

Supplement: S7 Fig — (A) Env staining on the surface of CEM.NKR-CCR5-sLTR-Luc cells infected with SIVmac239, SIVmac316, SIVsmE543-3, SIVsmE660-FL14 and SHIV AD8-EO was measured in the presence and absence of soluble CD4 (sCD4) (10 μg/ml) relative to non-specific staining of SHIV AD8-EO-infected cells (shaded). Antibody binding to SIV-infected (Gag+ CD4low) cells was detected with an AF647-conjugated anti-human IgG F(ab′)2. (B) ADCC responses (% RLU) were measured as the dose-dependent loss of luciferase activity from SIV-infected CEM.NKR-CCR5-sLTR-Luc cells after an 8-hour incubation with a rhesus macaque CD16+ NK cell line at a 10:1 E:T ratio and the indicated concentrations of each antibody in the presence and absence of sCD4 (10 μg/ml). The plotted values represent the mean and standard deviation (error bars) for triplicate wells and the dotted line indicates half-maximal ADCC. (TIF) [file ppat.1011407.s007.tif]
